# Supplementary material for: Discovery of [1,2,4]Triazole Derivatives as New Metallo-β-Lactamase Inhibitors
Source: Molecules. 2019 Dec 23;25(1):56. doi: 10.3390/molecules25010056 (PMC6982996; doi:10.3390/molecules25010056)
Supplement: Supplementary file 1 [file molecules-25-00056-s001.pdf]

Supporting Information

Article

Discovery of [1,2,4]Triazole Derivatives as New Metallo-β-Lactamase Inhibitors

Chen Yuan [1], Jie Yan [1], Chen Song [1], Fan Yang [1], Chao Li [1], Cheng Wang [1], Huiling Su [1], Wei Chen [1], Lijiao Wang [1], Zhouyu Wang [2], Shan Qian [1,*] and Lingling Yang [1,*]

[1] College of Food and Bioengineering, Xihua University, Chengdu 610039, China; 18380460102@163.com (C.Y.); 18328072545@163. Com (J.Y.); sc1475778467@163.com (C.S.); 18228209592@163.com (F.Y.); 18408240706@163.com (C.L.); 15182011493@163.com (C.W.); 15982178237@163.com (H.S.); CW13980419963@163.com (W.C.); wanglijiao@mail.xhu.edu.cn (L.W.)

[2] College of Science, Xihua University, Chengdu 610039, China; zhouyuwang77@gmail.com

* Correspondence: qians33@163.com (S.Q.); yangll0808@sina.com (L.Y.); Tel.: +86-28-7725898 (L.Y.)

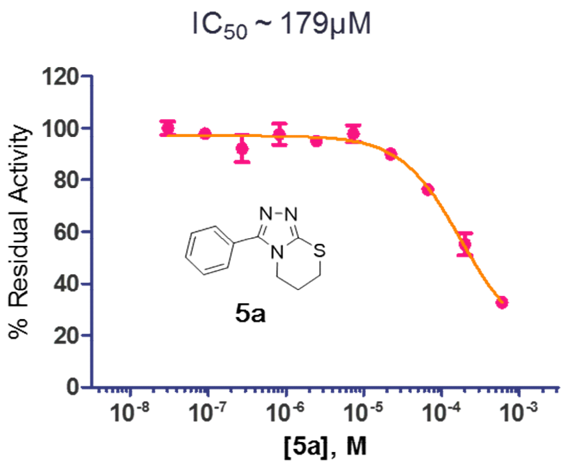

Figure S1. The IC_{50} curve of 5a against VIM-2. The IC_{50} value is about 179 μM.

^{1}H NMR and ^{13}CNMR spectra of target compounds

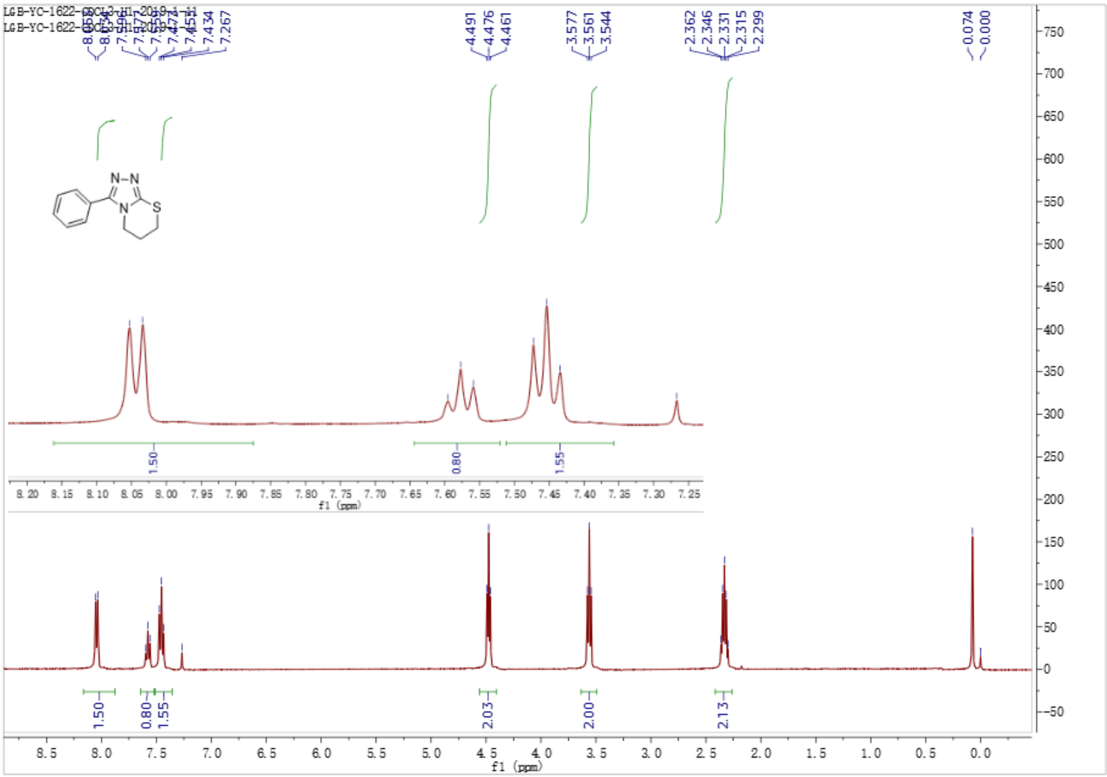

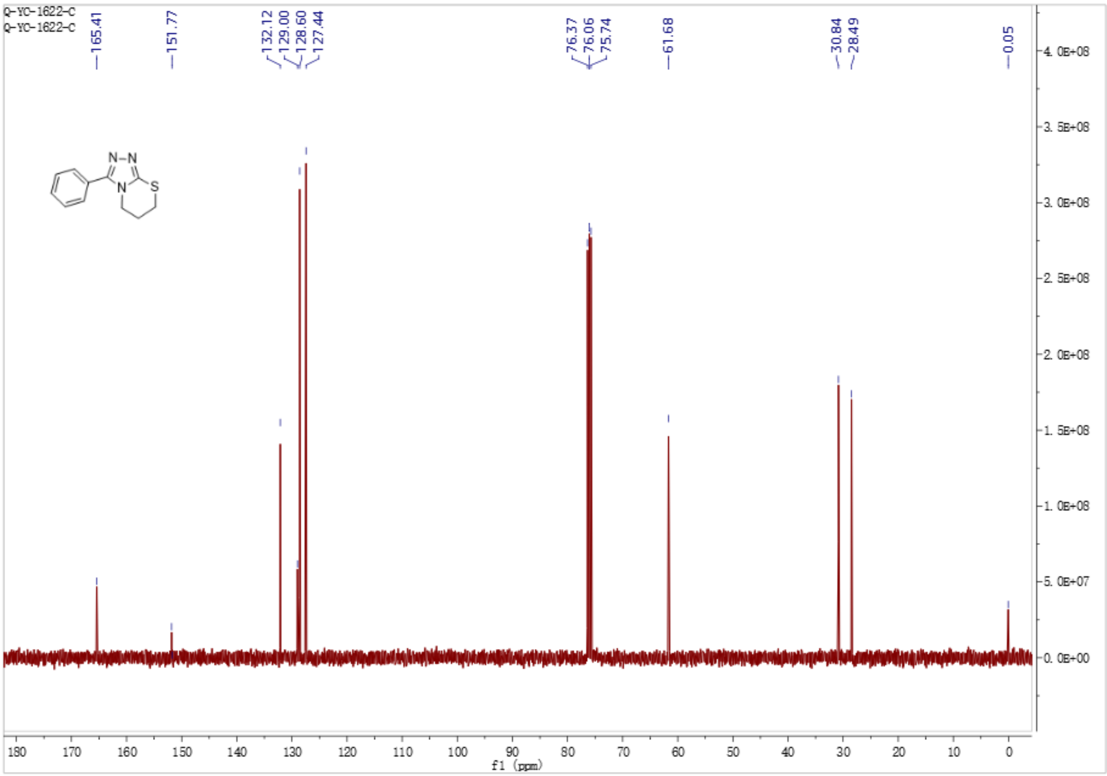

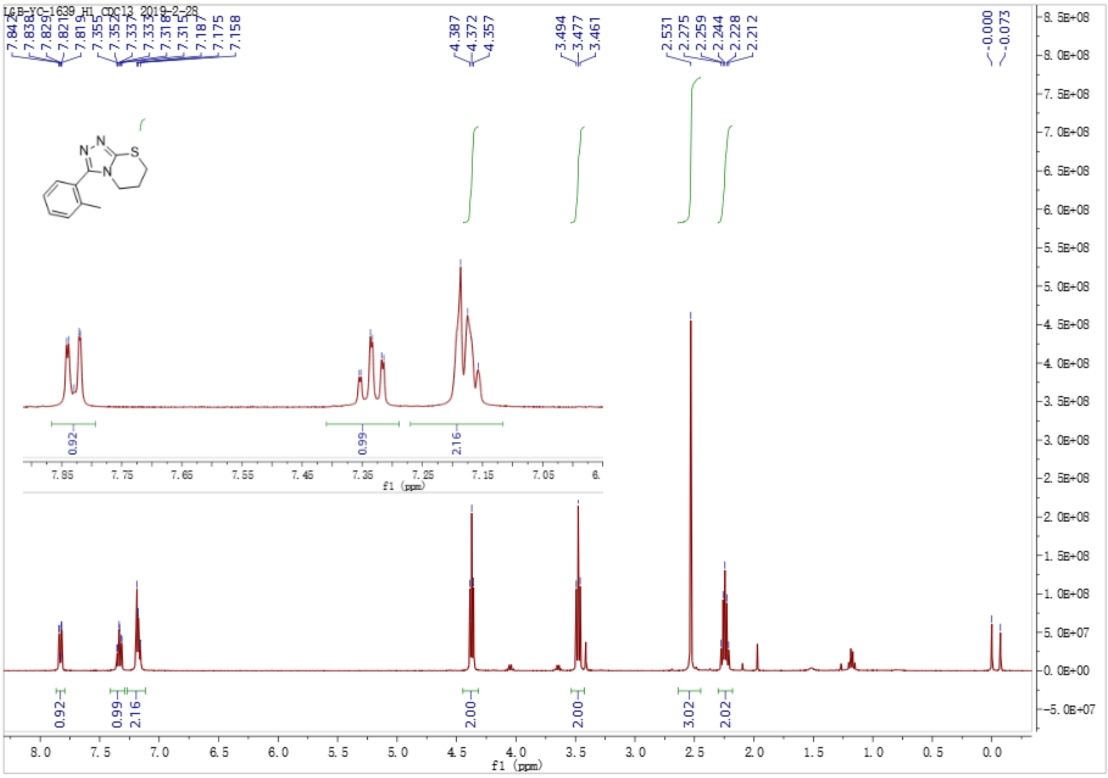
LGB-YC-1639_H1_CDCl3_2019-2-28
7.842
7.838
7.829
7.821
7.819
7.355
7.353
7.337
7.333
7.318
7.315
7.187
7.175
7.158
4.387
4.372
4.357
3.494
3.477
3.461
2.531
2.275
2.259
2.244
2.228
2.212
0.000
-0.073
N
N
N
S
0.92
0.99
2.16
2.00
2.00
3.02
2.02
f1 (ppm)
f1 (ppm)

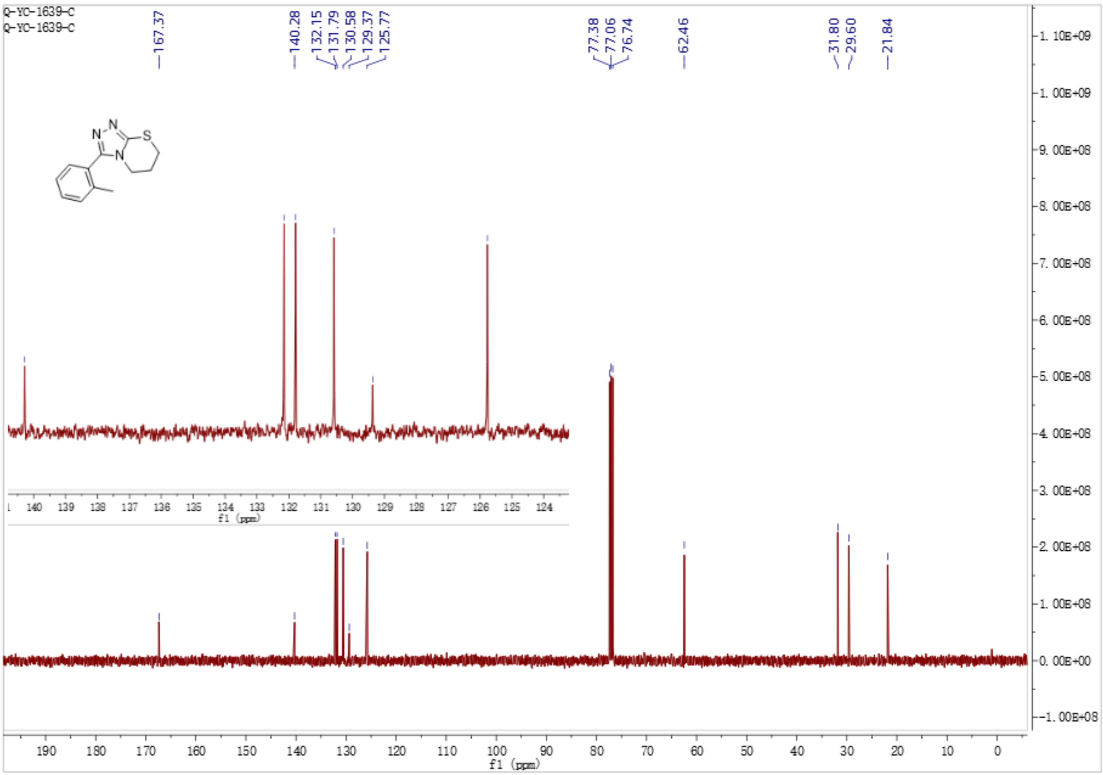
Q-YC-1639-C
Q-YC-1639-C
167.37
140.28
132.15
131.79
130.58
129.37
125.77
77.38
77.06
76.74
62.46
31.80
29.60
21.84
N
N
N
S
f1 (ppm)
f1 (ppm)

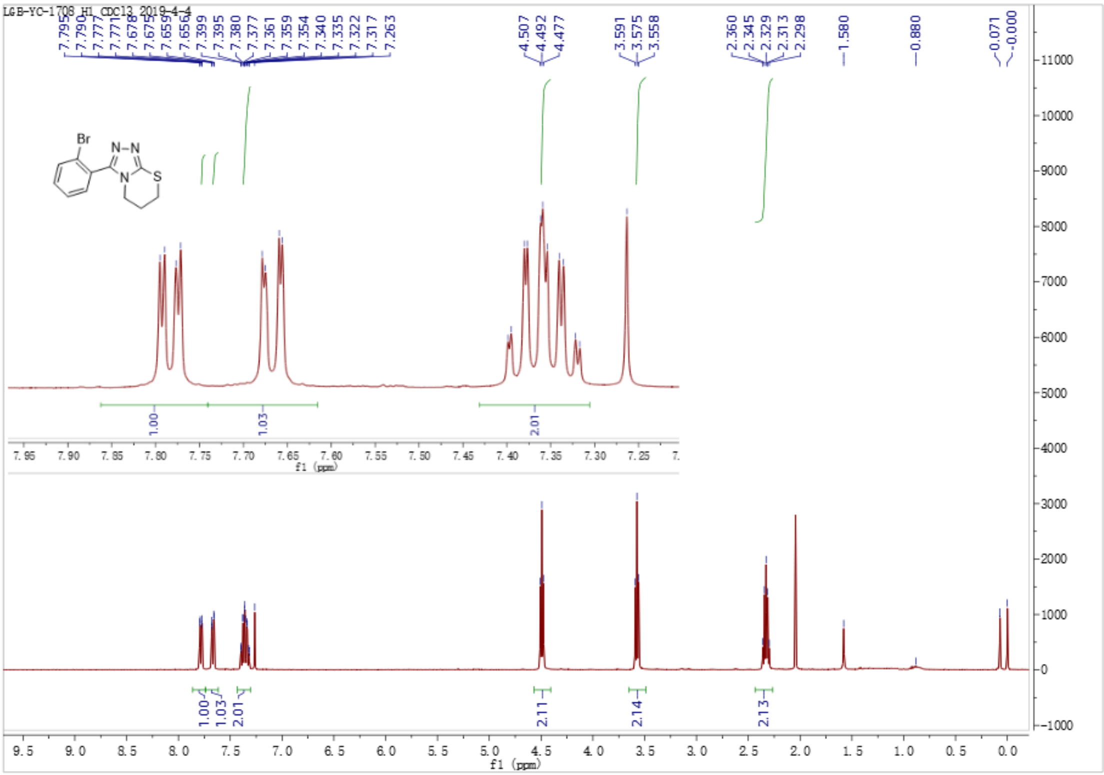
LGB-YC-1708_H1_CDCl3_2019-4-4
f1 (ppm)

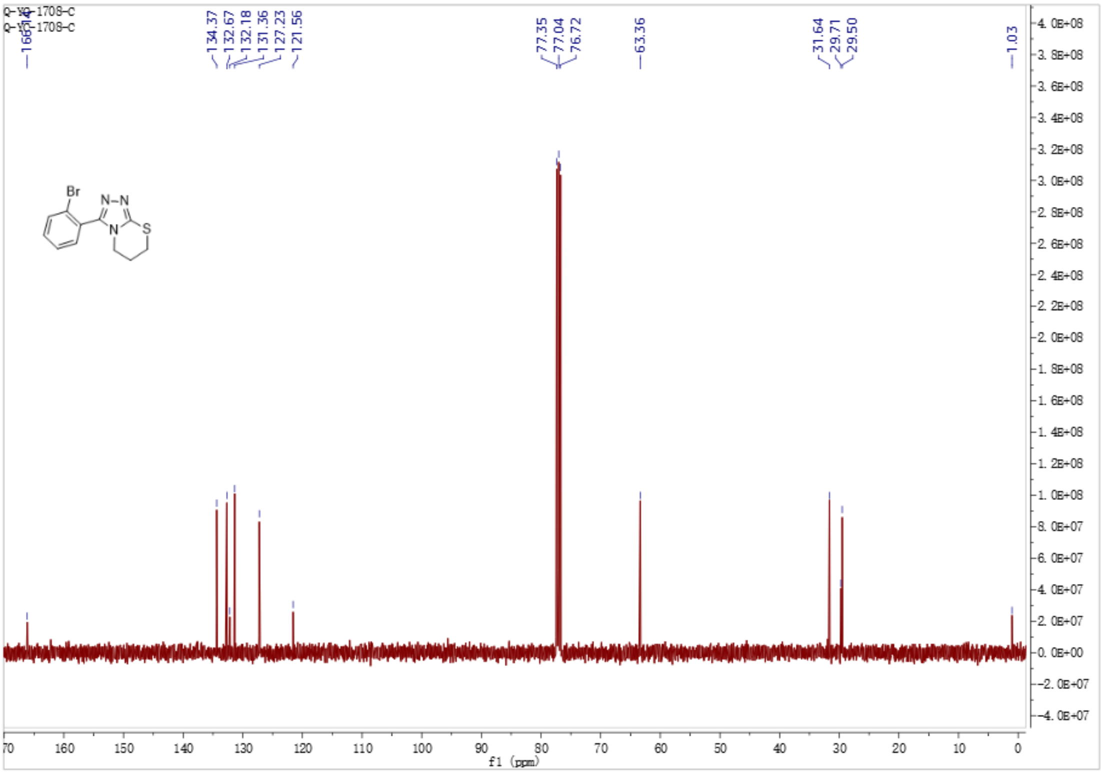
Q-YC-1708-C
f1 (ppm)

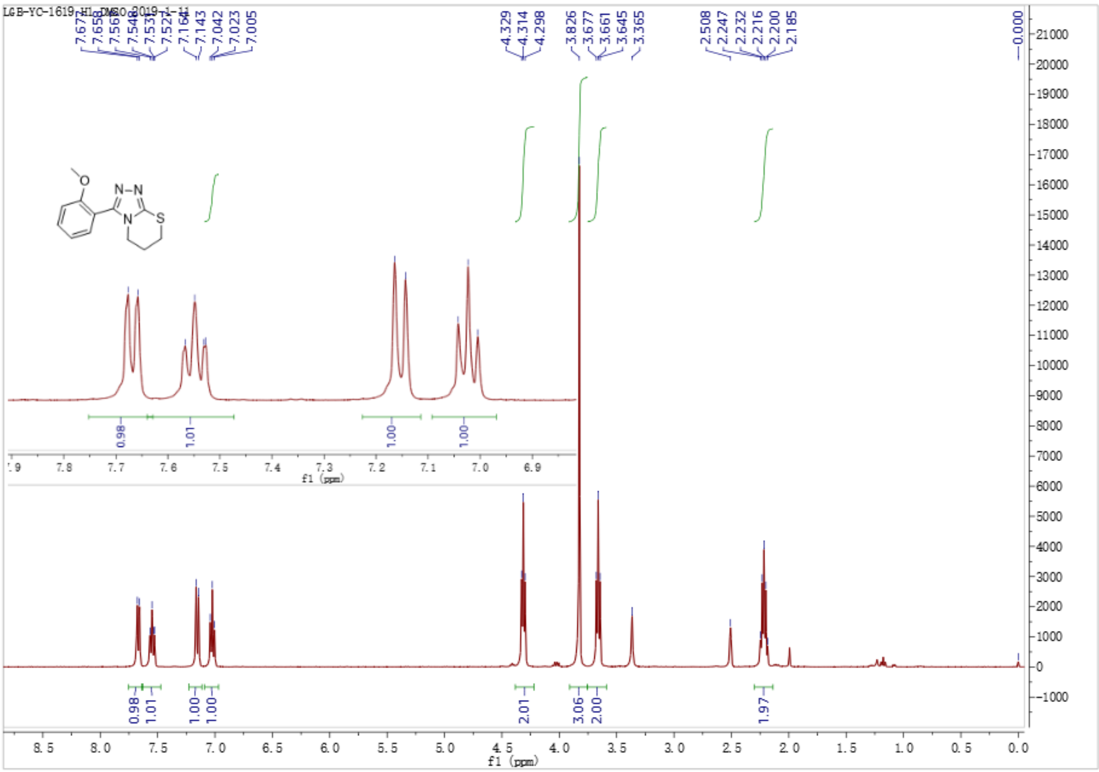
LGB-YC-1619-H1-DMSO-2019-1-11
7.677
7.658
7.563
7.548
7.531
7.527
7.164
7.143
7.042
7.023
7.005
4.329
4.314
4.298
3.826
3.677
3.661
3.645
3.365
2.508
2.247
2.232
2.216
2.200
2.185
0.000
0.98
1.01
1.00
1.00
2.01
3.06
2.00
1.97
f1 (ppm)

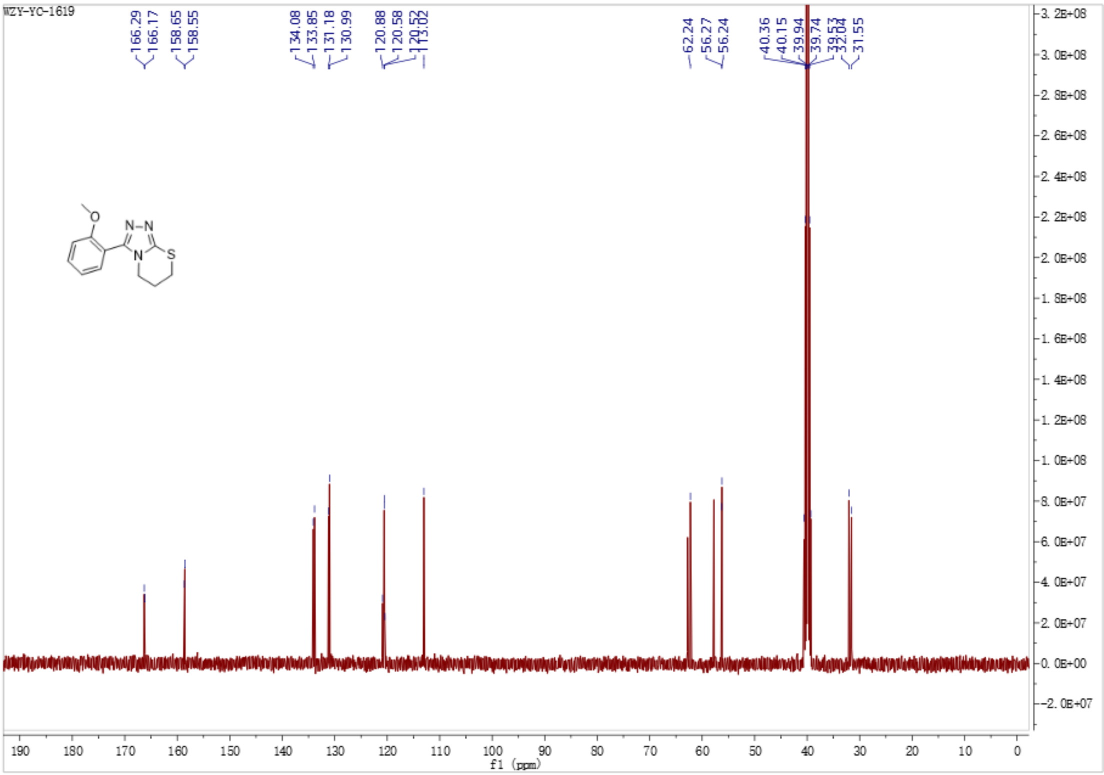
WZY-YC-1619
166.29
166.17
158.65
158.55
134.08
133.85
131.18
130.99
120.88
120.58
120.52
113.02
62.24
56.27
56.24
40.36
40.15
39.94
39.74
39.53
32.04
31.55
f1 (ppm)

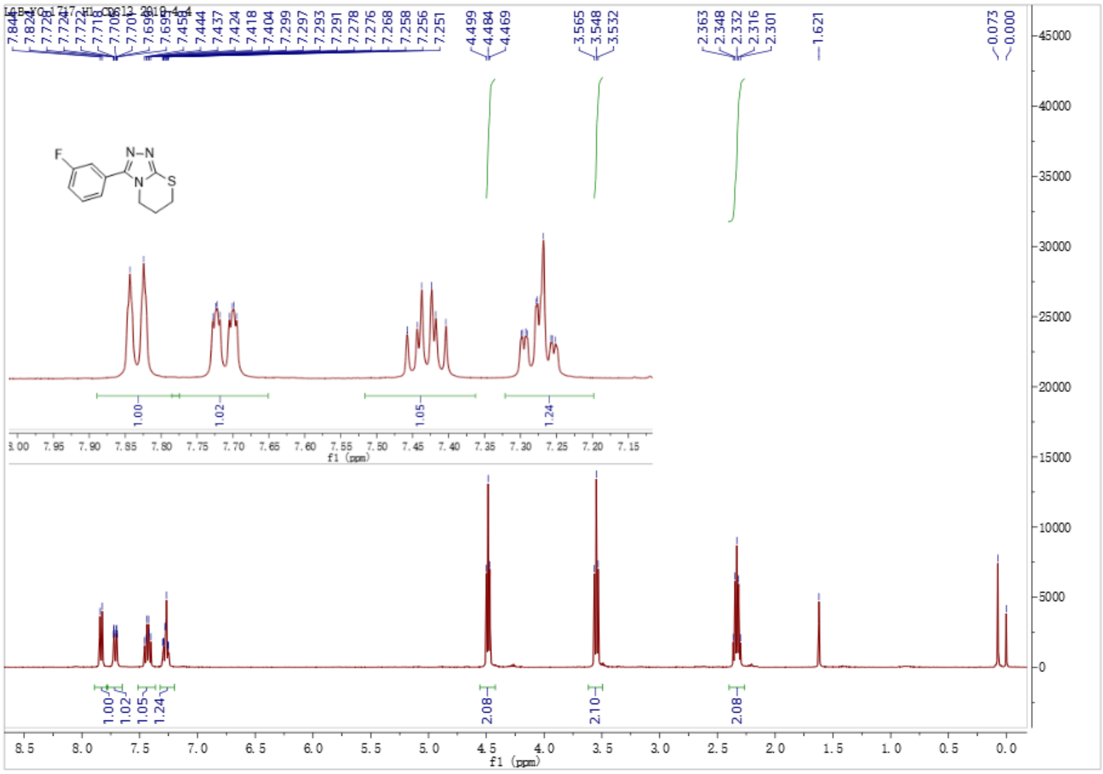

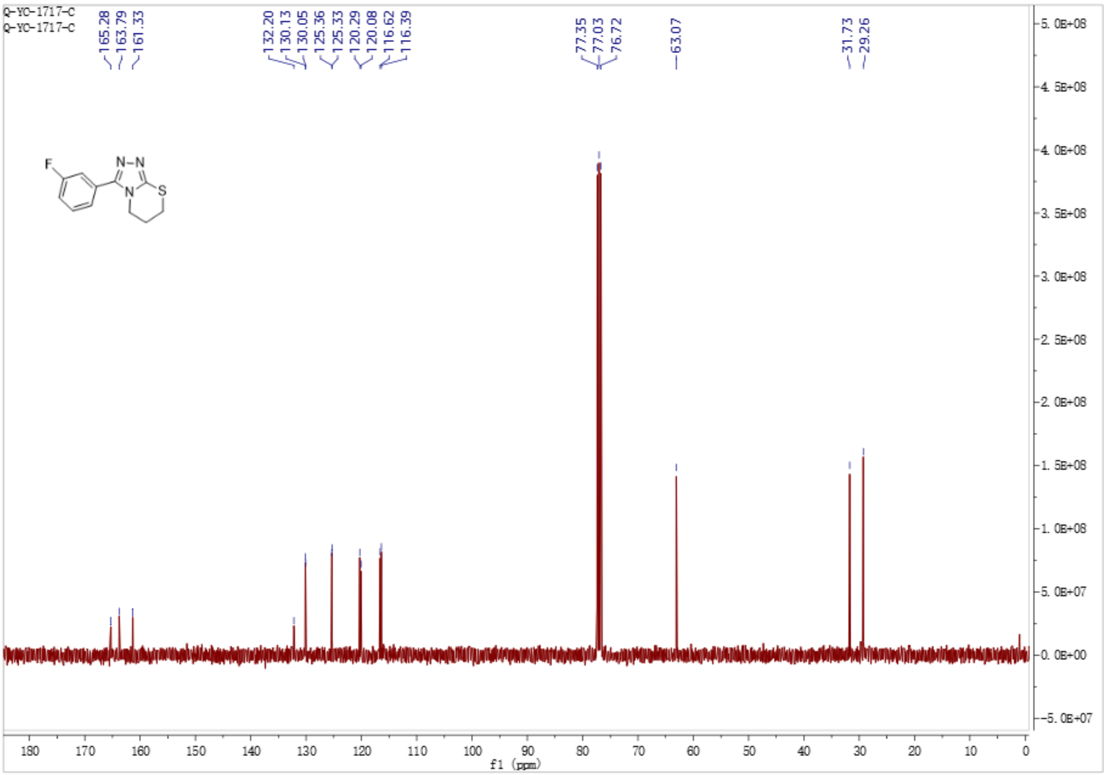

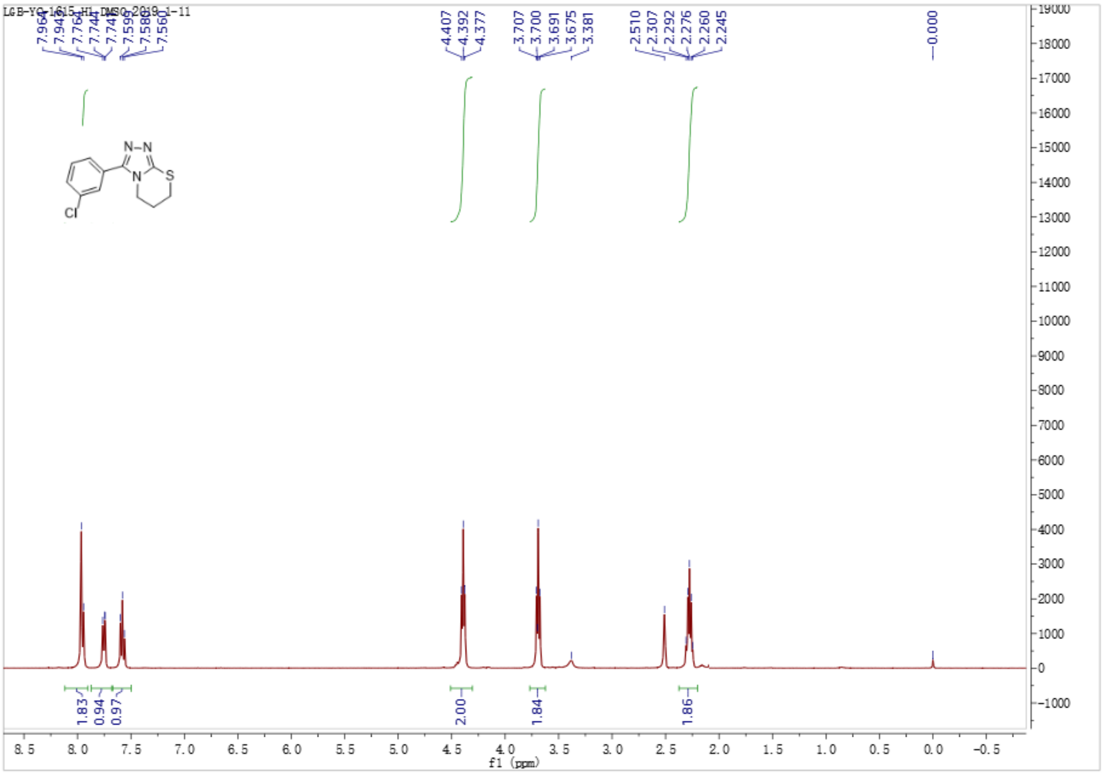
LGB-YG-1615_H1_DMSO_2019_1-11
7.964
7.941
7.764
7.744
7.741
7.599
7.580
7.560
4.407
4.392
4.377
3.707
3.700
3.691
3.675
3.381
2.510
2.307
2.292
2.276
2.260
2.245
0.000
1.83
0.94
0.97
2.00
1.84
1.86
f1 (ppm)
N-N
N
S
Cl

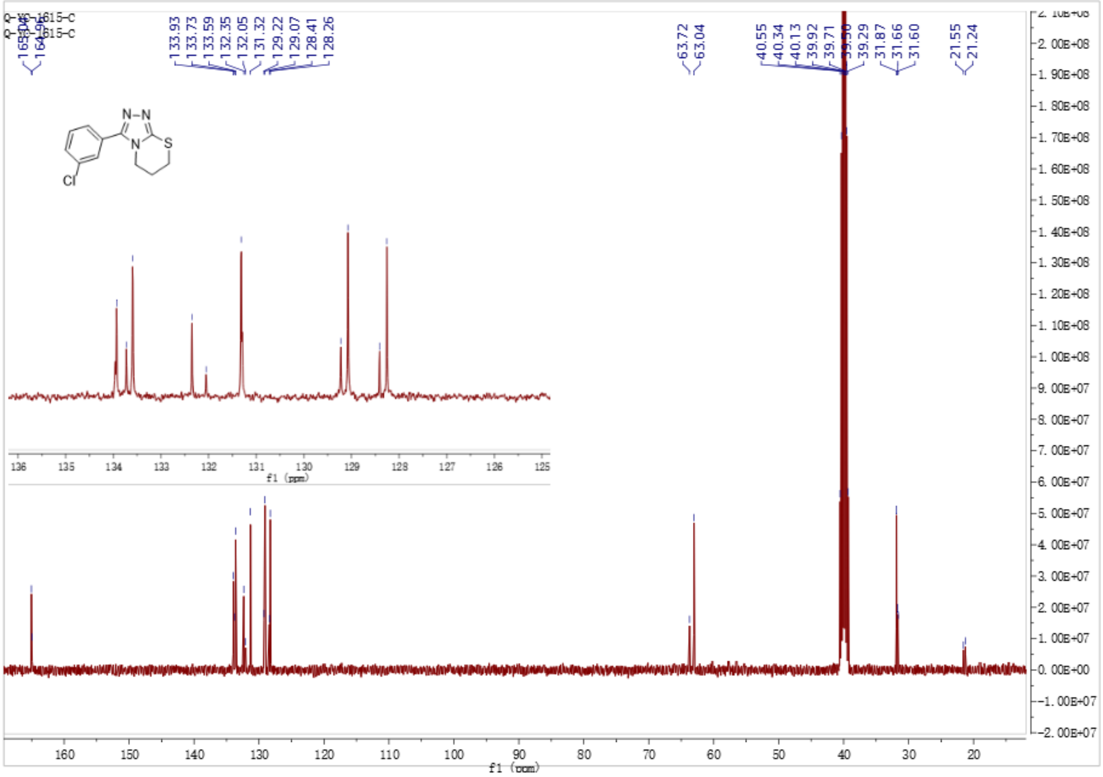
Q-YG-1615-C
165.04
164.96
133.93
133.73
133.59
132.35
132.05
131.32
129.22
129.07
128.41
128.26
63.72
63.04
40.55
40.34
40.13
39.92
39.71
39.50
39.29
31.87
31.66
31.60
21.55
21.24
f1 (ppm)
N-N
N
S
Cl

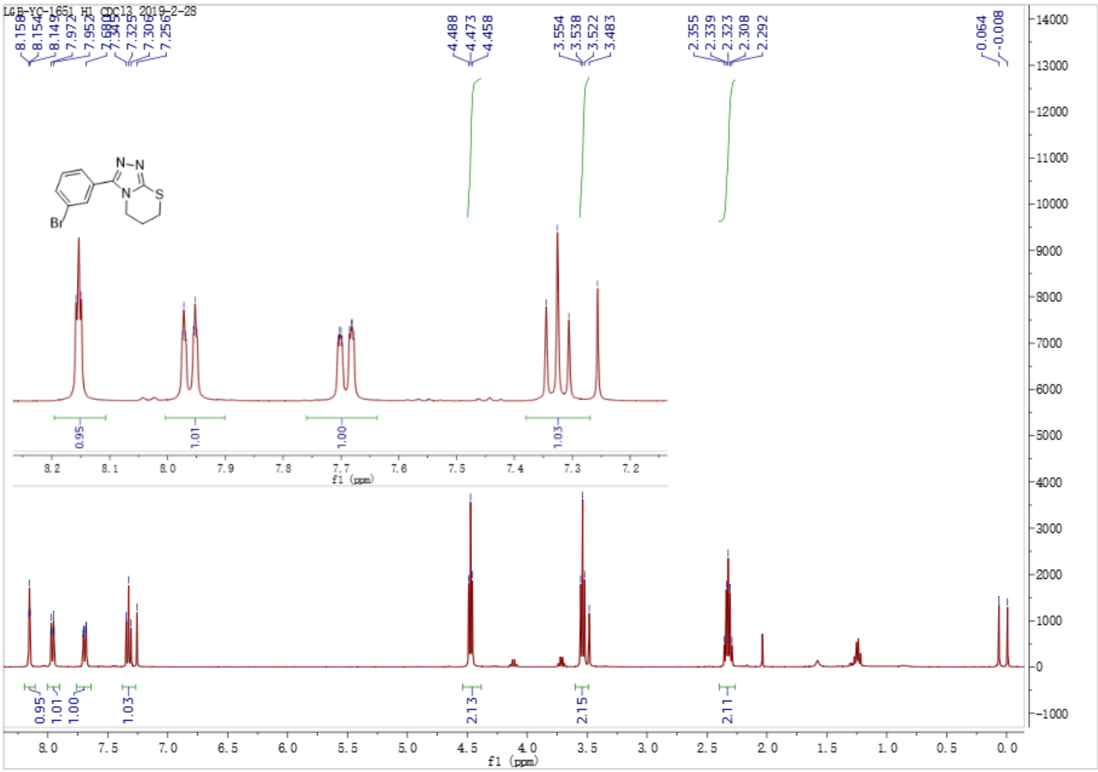

8.158
8.154
8.149
7.972
7.952
7.325
7.306
7.256
4.488
4.473
4.458
3.554
3.538
3.522
3.483
2.355
2.339
2.323
2.308
2.292
0.064
-0.008
N
N
N
S
Br
0.95
1.01
1.00
1.03
2.13
2.15
2.11
f1 (ppm)

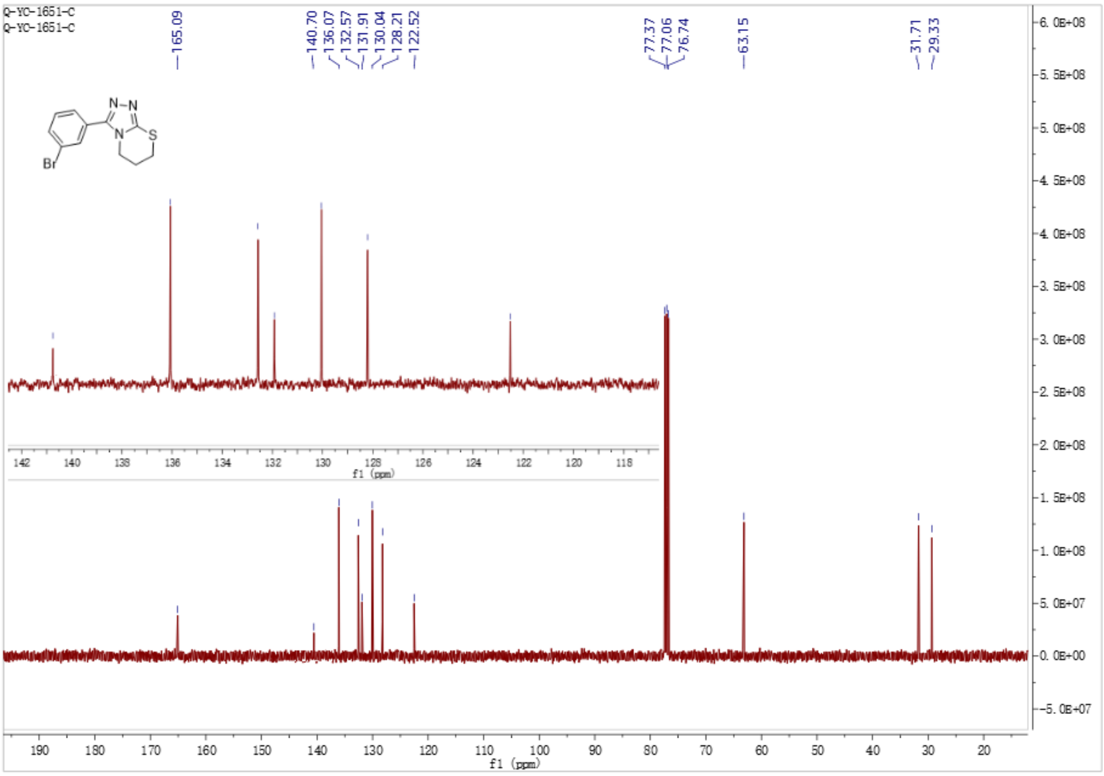

Q-YC-1651-C
Q-YC-1651-C
165.09
140.70
136.07
132.57
131.91
130.04
128.21
122.52
77.37
77.06
76.74
63.15
31.71
29.33
N
N
N
S
Br
f1 (ppm)

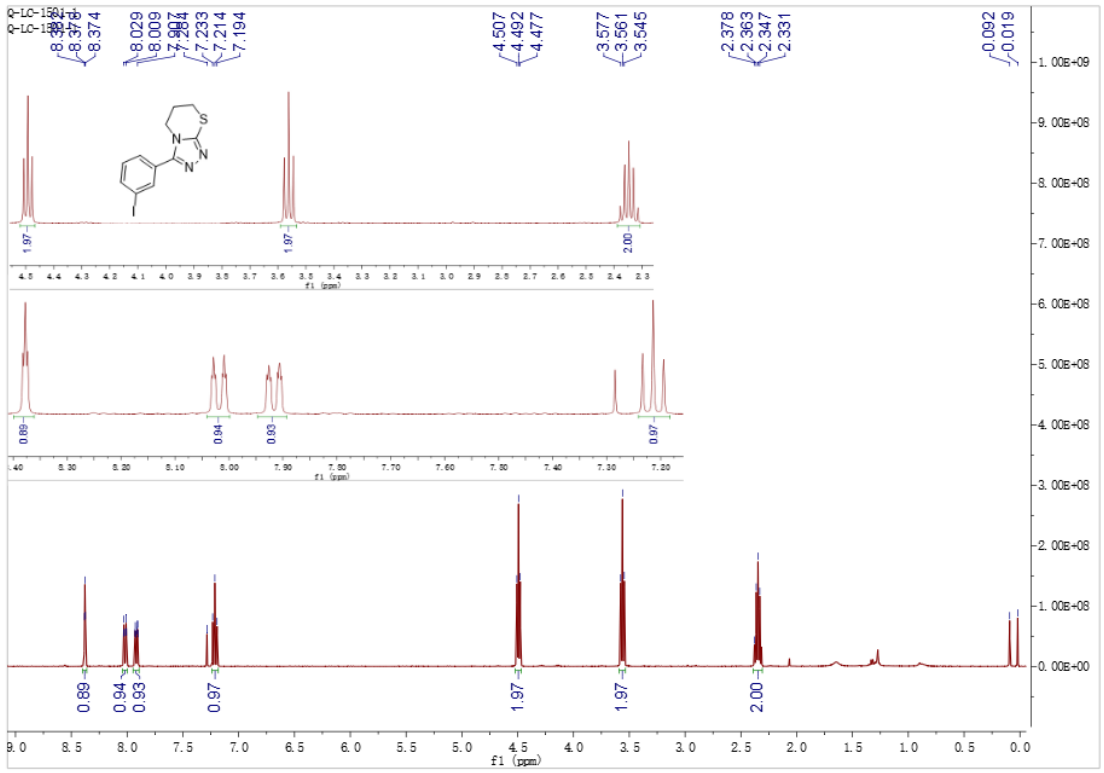
Q-LC-1501-1
8.378
8.374
8.029
8.009
7.233
7.214
7.194
4.507
4.492
4.477
3.577
3.561
3.545
2.378
2.363
2.347
2.331
0.092
0.019
0.89
0.94
0.93
0.97
1.97
1.97
2.00
f1 (ppm)

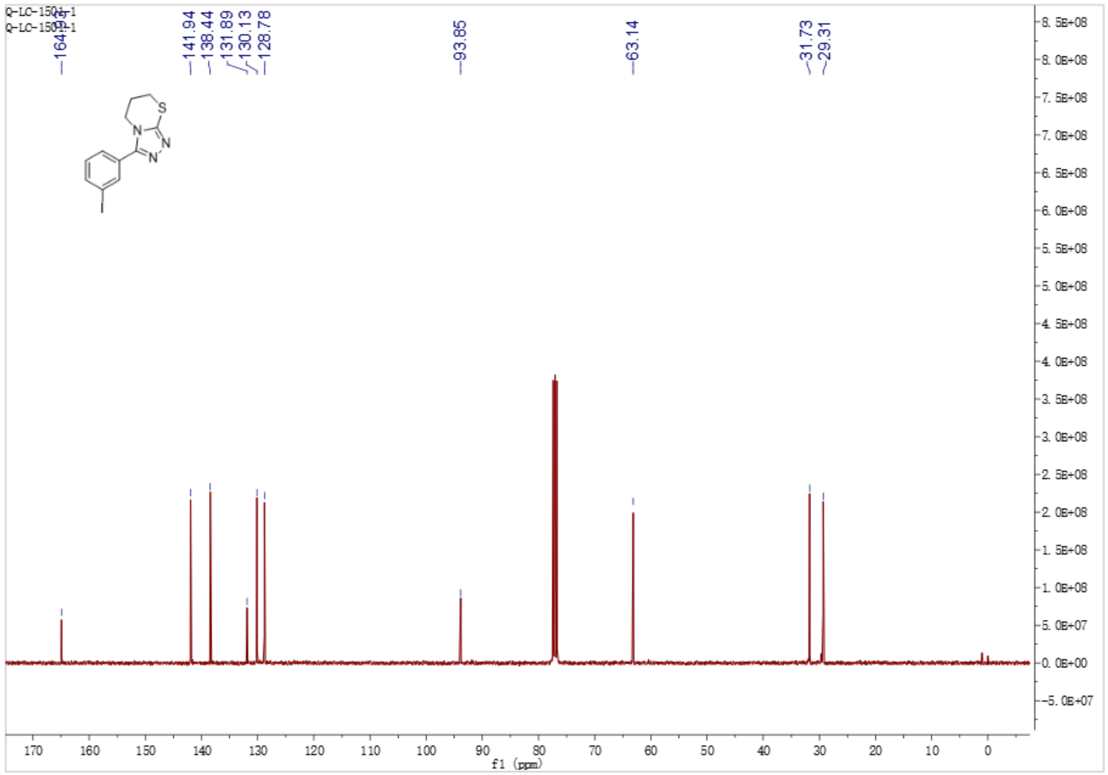
Q-LC-1501-1
141.94
138.44
131.89
130.13
128.78
93.85
63.14
31.73
29.31
f1 (ppm)

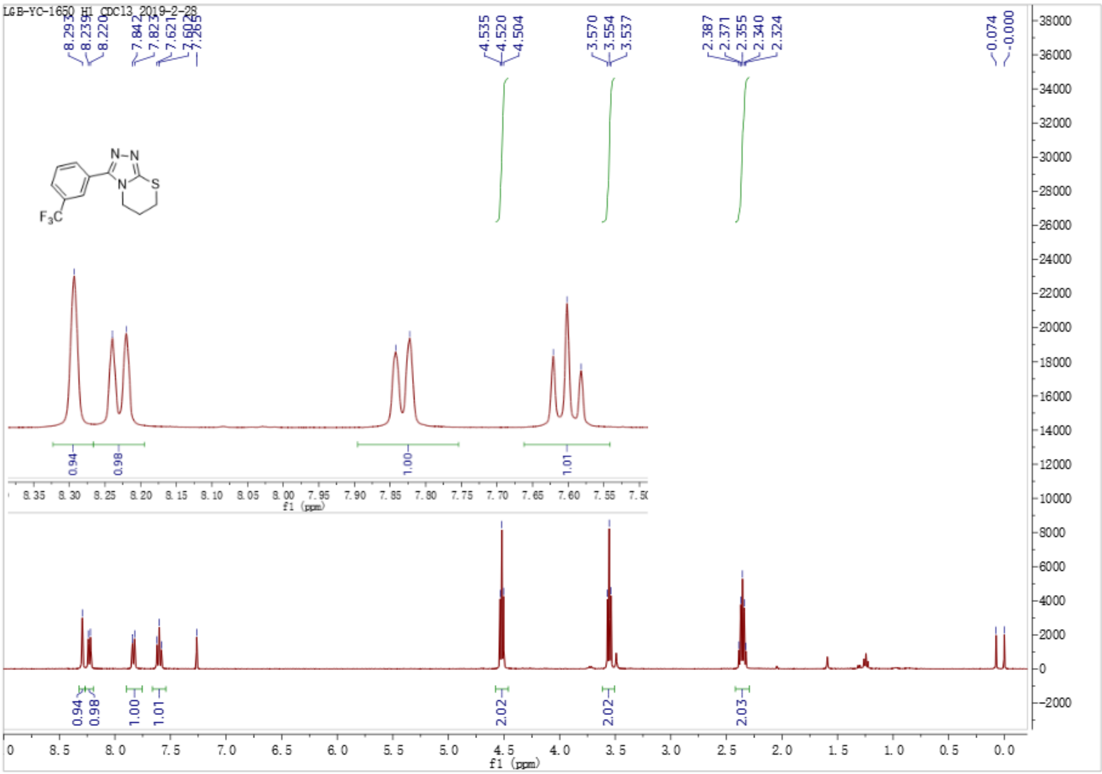

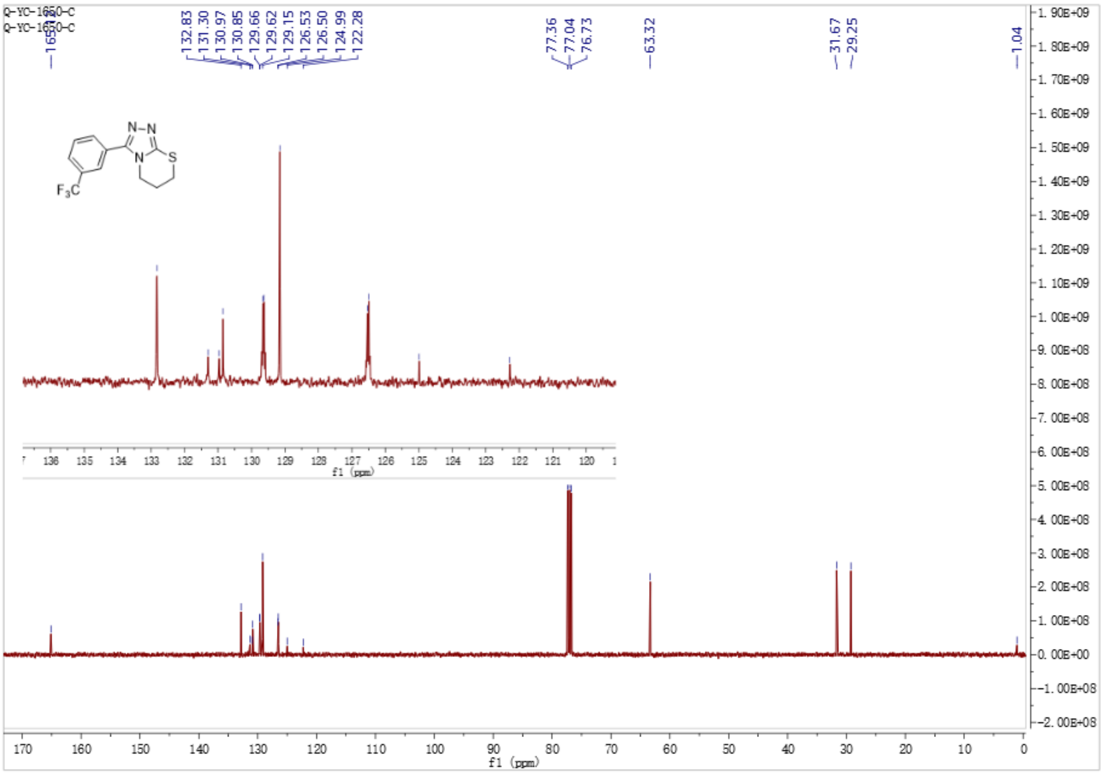

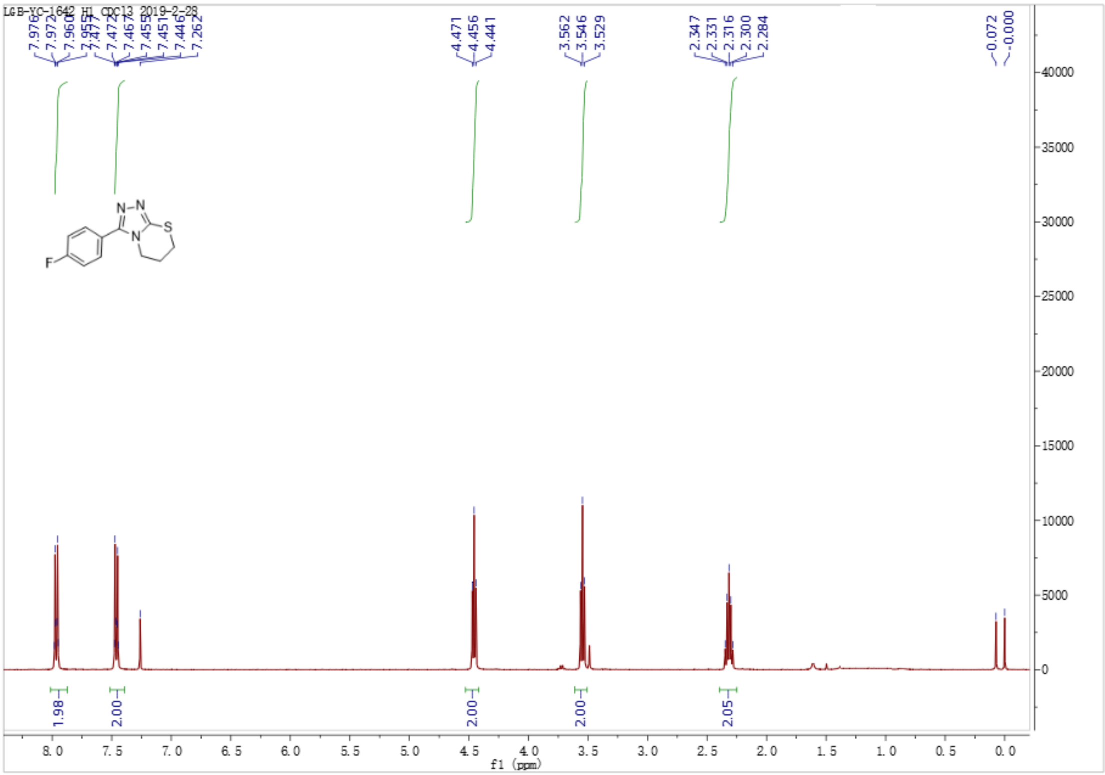
LGB-YC-1642 H1 CDCl3 2019-2-28
7.978
7.972
7.960
7.955
7.477
7.472
7.467
7.455
7.451
7.446
7.262
4.471
4.456
4.441
3.562
3.546
3.529
2.347
2.331
2.316
2.300
2.284
0.072
-0.000
N–N
S
N
F
1.98
2.00
2.00
2.00
2.05
f1 (ppm)

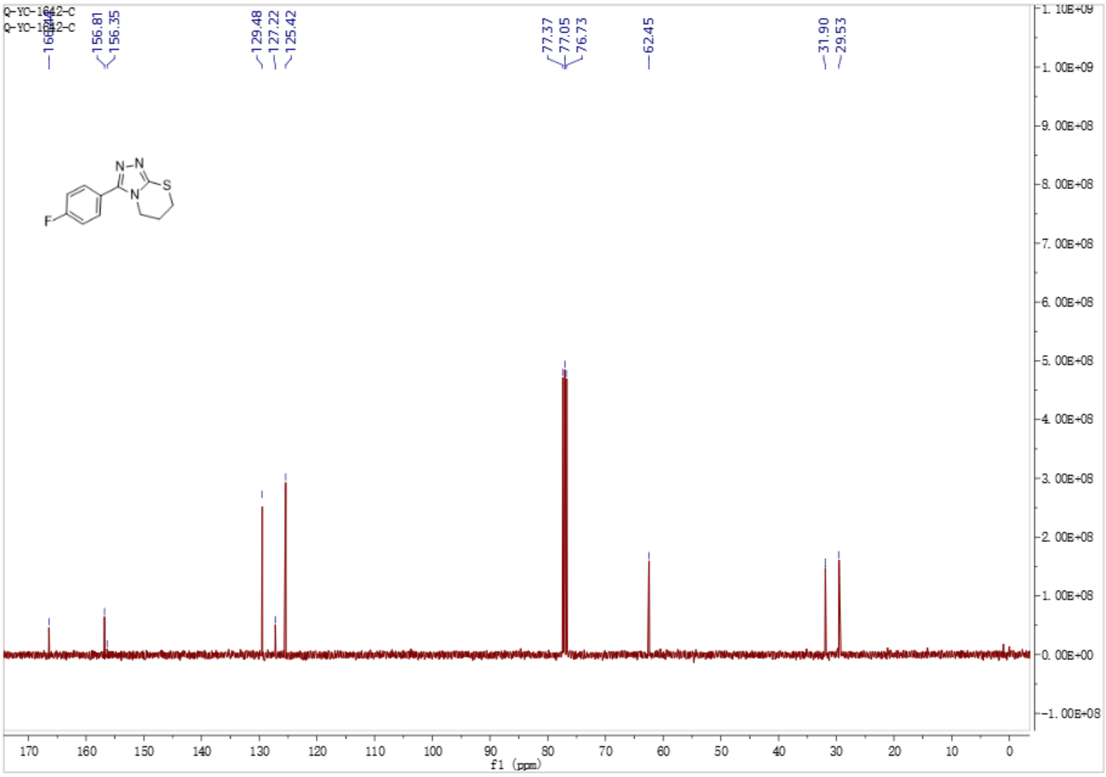
Q-YC-1642-C
156.81
156.35
129.48
127.22
125.42
77.37
77.05
76.73
62.45
31.90
29.53
N–N
S
N
F
f1 (ppm)

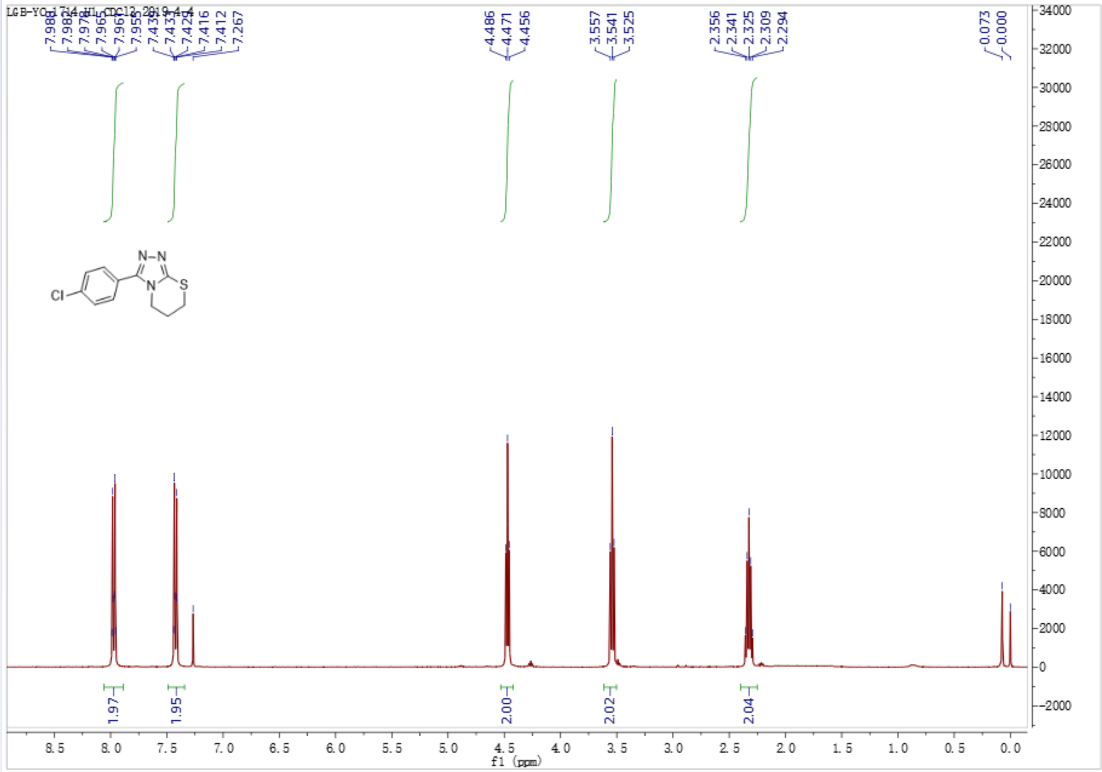
7.988
7.983
7.976
7.965
7.961
7.955
7.439
7.435
7.426
7.416
7.412
7.267
4.486
4.471
4.456
3.557
3.541
3.525
2.356
2.341
2.325
2.309
2.294
0.073
0.000
N–N
S
N
Cl
1.97
1.95
2.00
2.02
2.04
f1 (ppm)

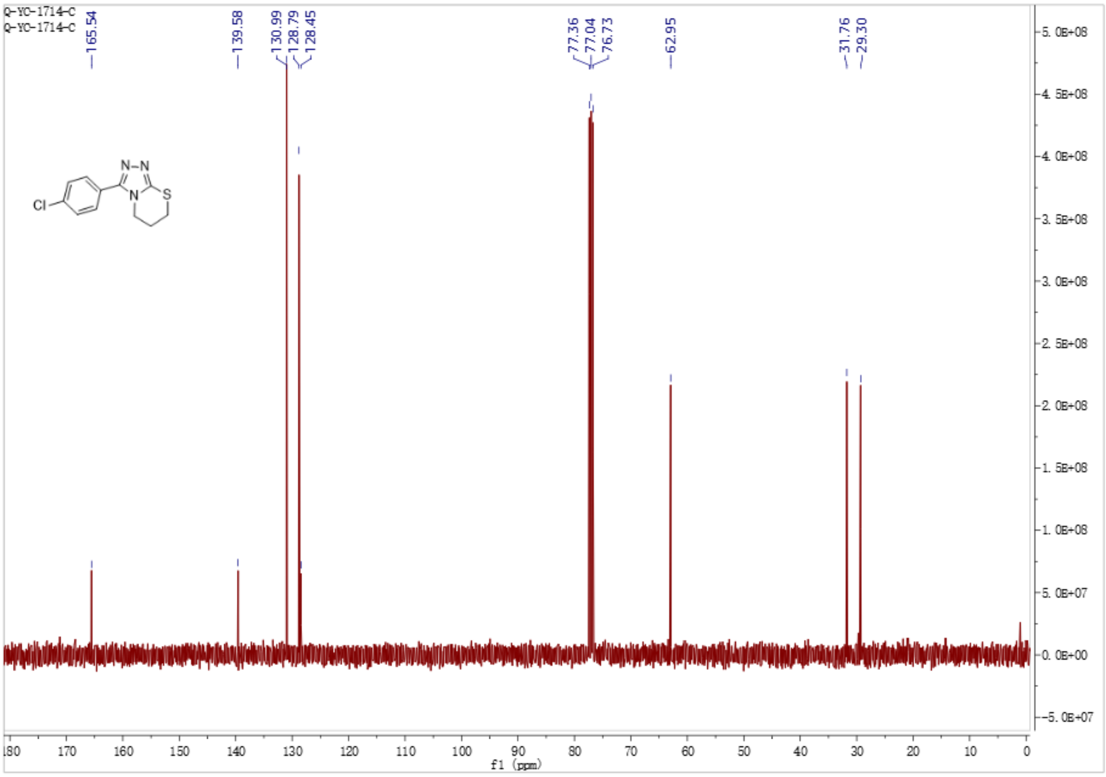
Q-YC-1714-C
Q-YC-1714-C
165.54
139.58
130.99
128.79
128.45
77.36
77.04
76.73
62.95
31.76
29.30
N–N
S
N
Cl
f1 (ppm)

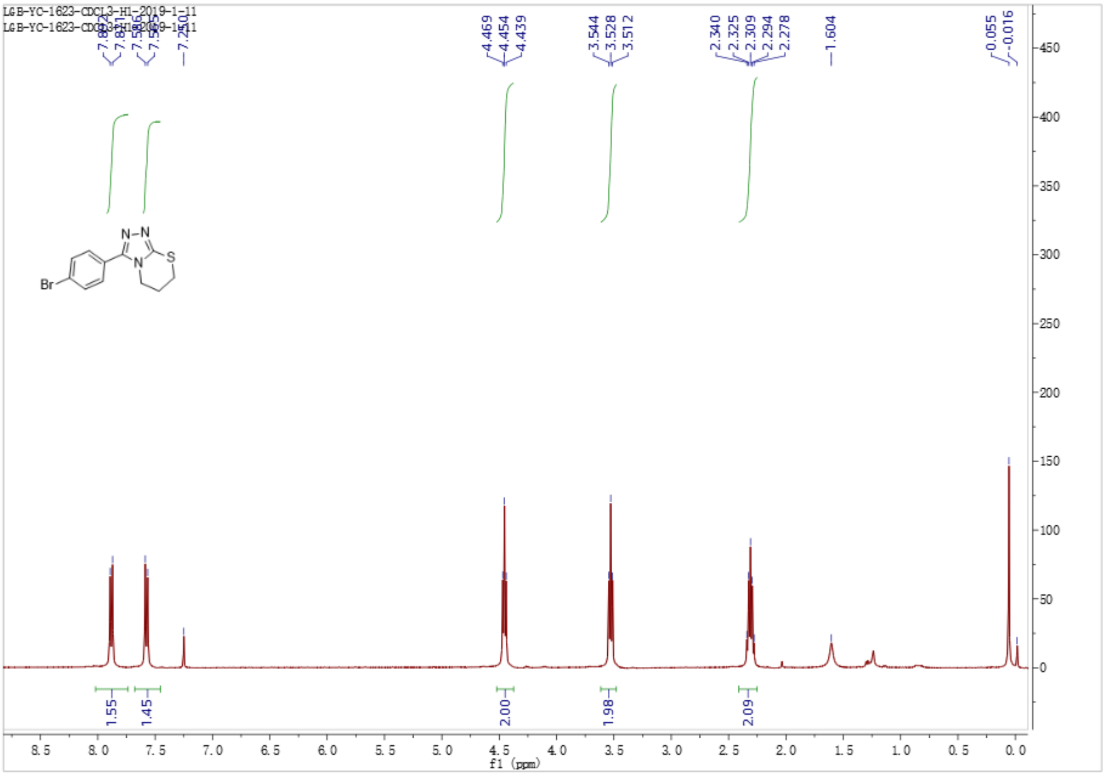
LGB-YC-1623-CDCL3-H1-2019-1-11
4.469
4.454
4.439
3.544
3.528
3.512
2.340
2.325
2.309
2.294
2.278
1.604
0.055
-0.016
1.55
1.45
2.00
1.98
2.09
f1 (ppm)
Br
N–N
N
S

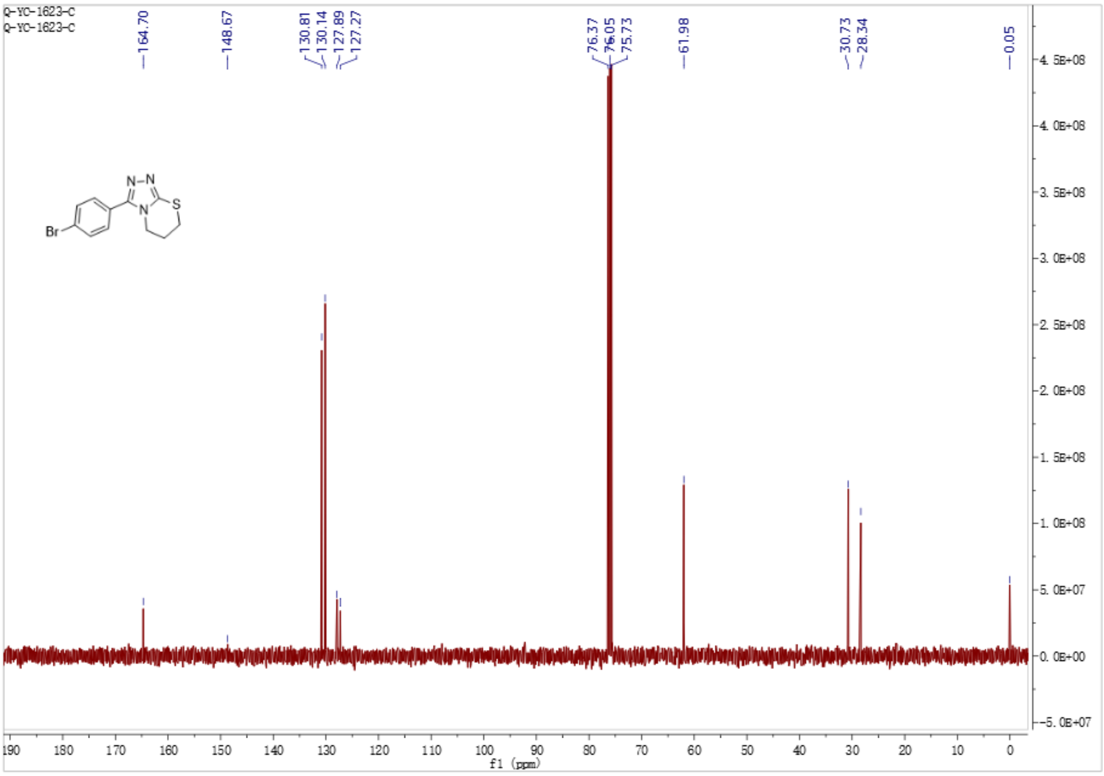
Q-YC-1623-C
164.70
148.67
130.81
130.14
127.89
127.27
76.37
76.05
75.73
61.98
30.73
28.34
0.05
f1 (ppm)
Br
N–N
N
S

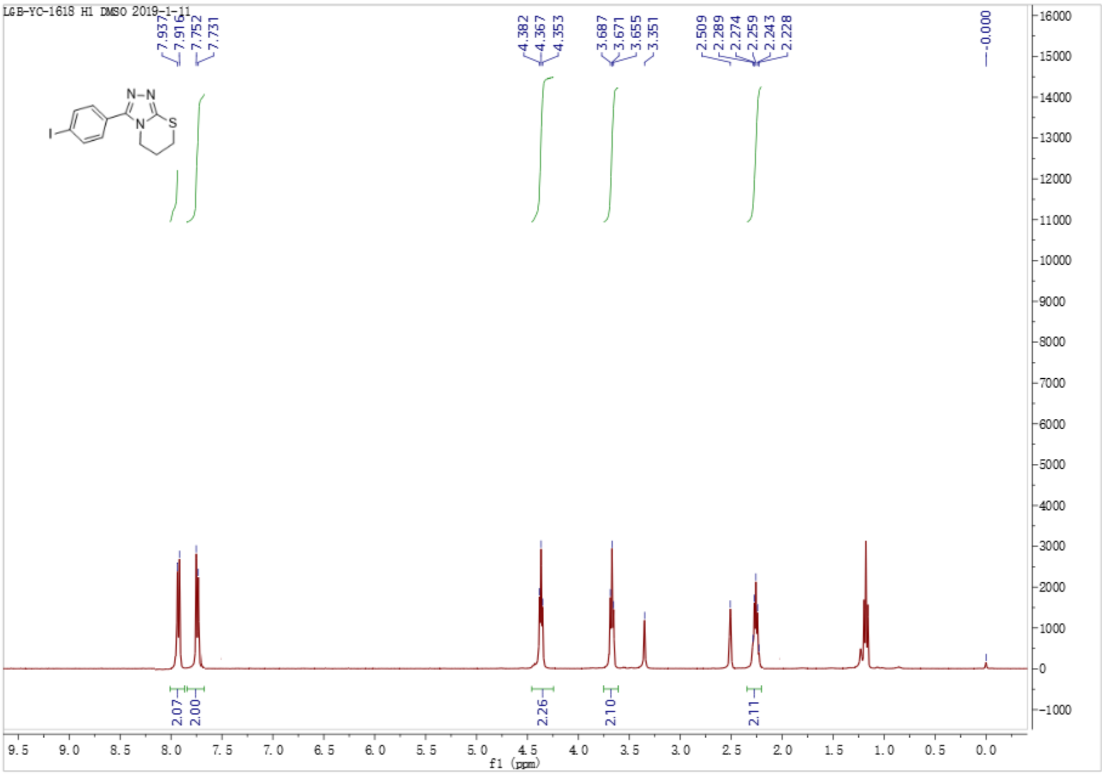
LGB-YC-1618 H1 DMSO 2019-1-11
7.937
7.916
7.752
7.731
4.382
4.367
4.353
3.687
3.671
3.655
3.351
2.509
2.289
2.274
2.259
2.243
2.228
-0.000
2.07
2.00
2.26
2.10
2.11
f1 (ppm)

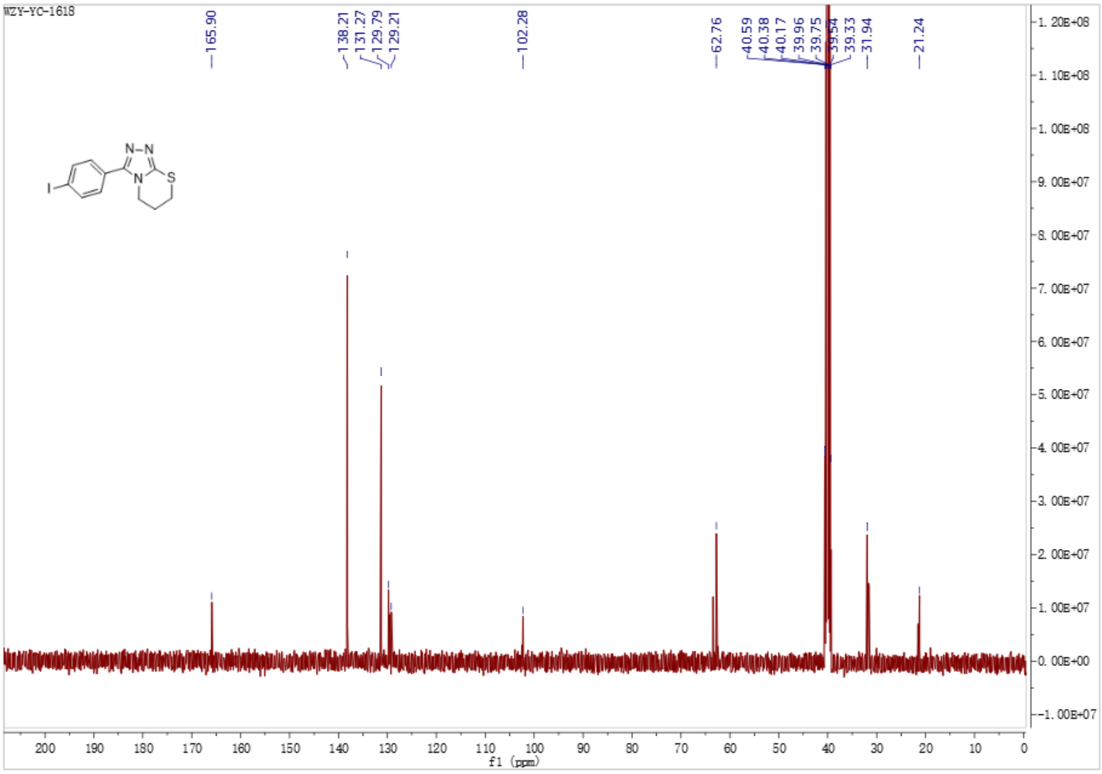
WZY-YC-1618
165.90
138.21
131.27
129.79
129.21
102.28
62.76
40.59
40.38
40.17
39.96
39.75
39.54
39.33
31.94
21.24
f1 (ppm)

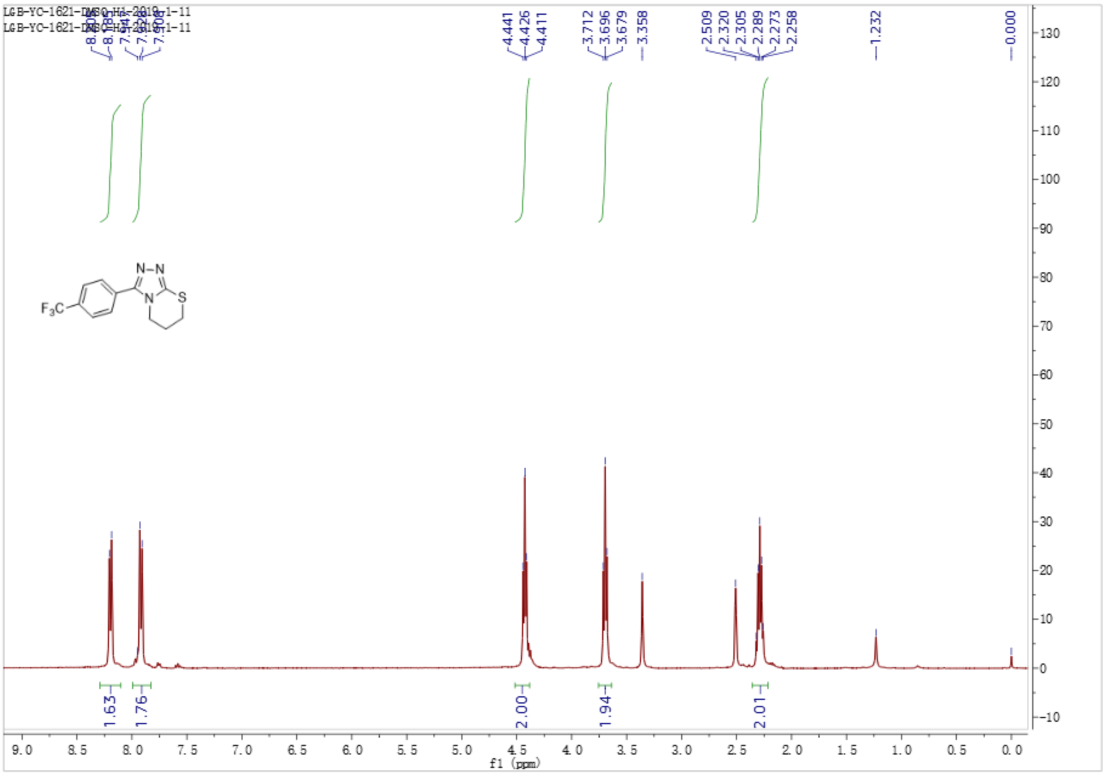
LGB-YC-1621-DMSO-H1-2019-1-11
4.441
4.426
4.411
3.712
3.696
3.679
3.358
2.509
2.320
2.305
2.289
2.273
2.258
1.232
0.000
1.63
1.76
2.00
1.94
2.01
f1 (ppm)

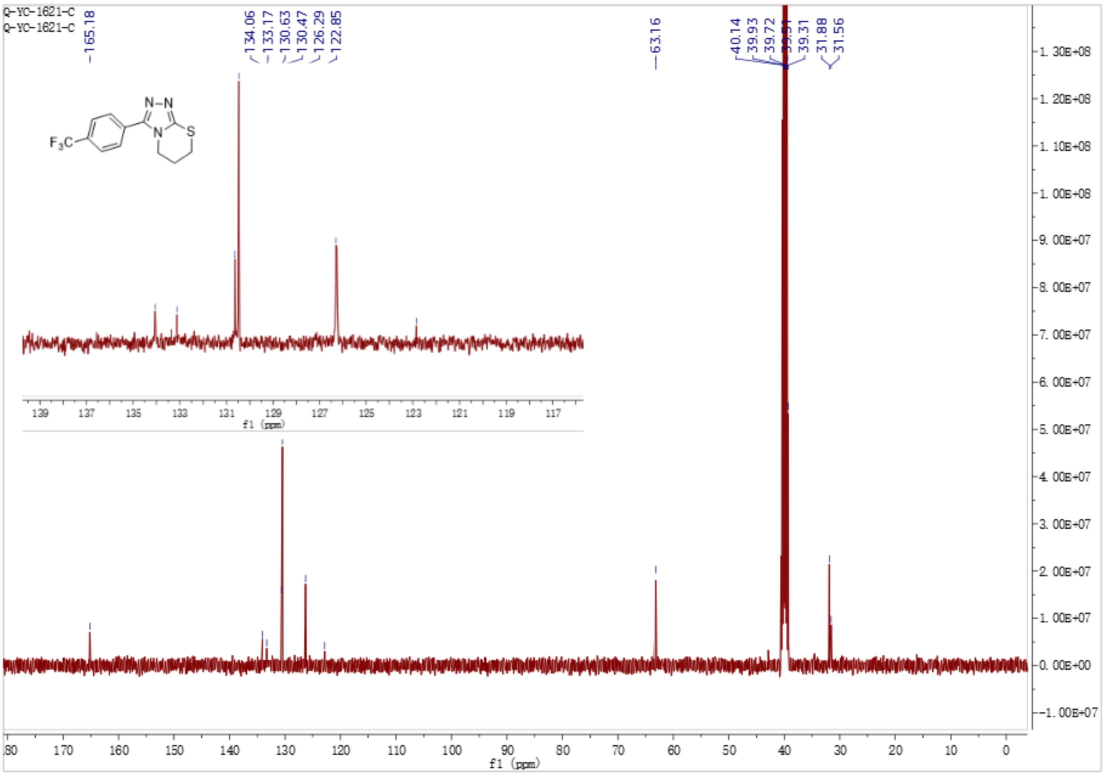
Q-YC-1621-C
165.18
134.06
133.17
130.63
130.47
126.29
122.85
63.16
40.14
39.93
39.72
39.31
31.88
31.56
f1 (ppm)

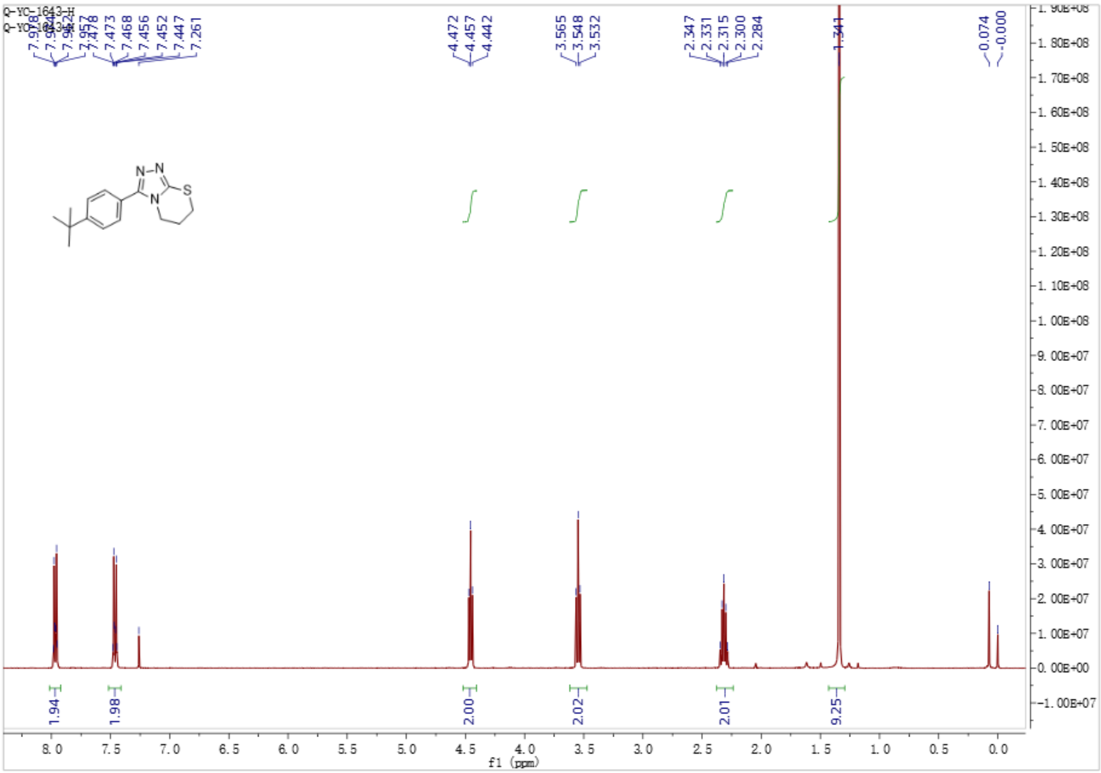

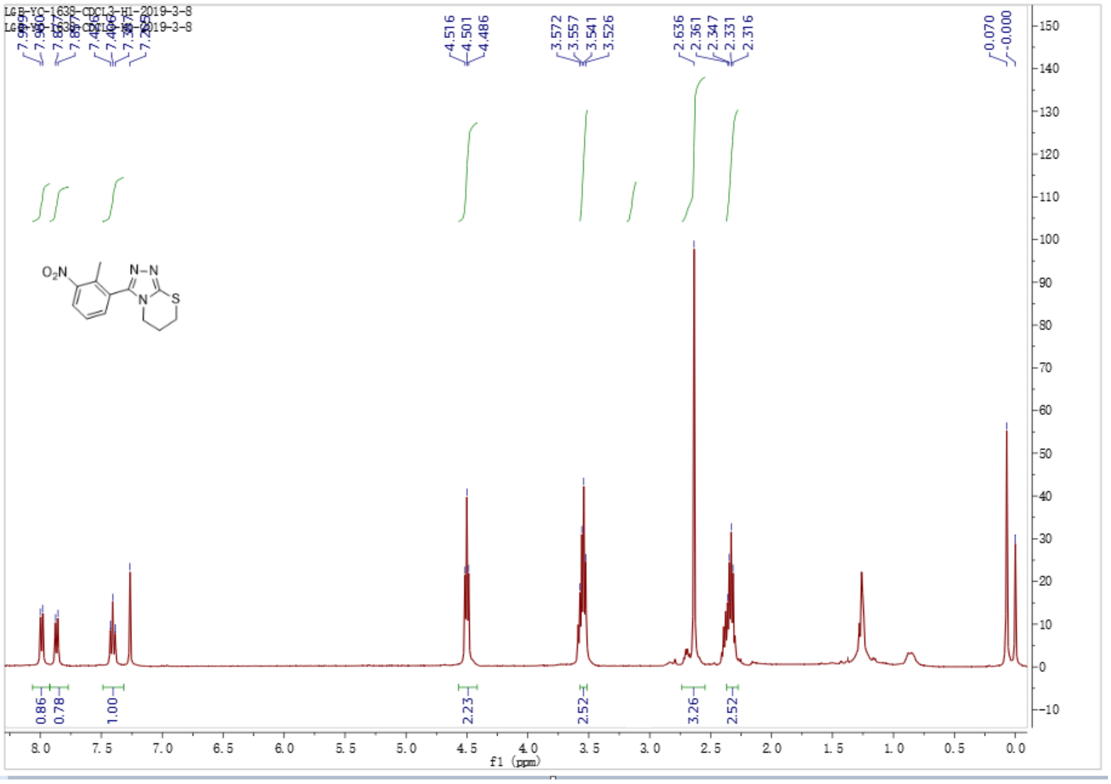

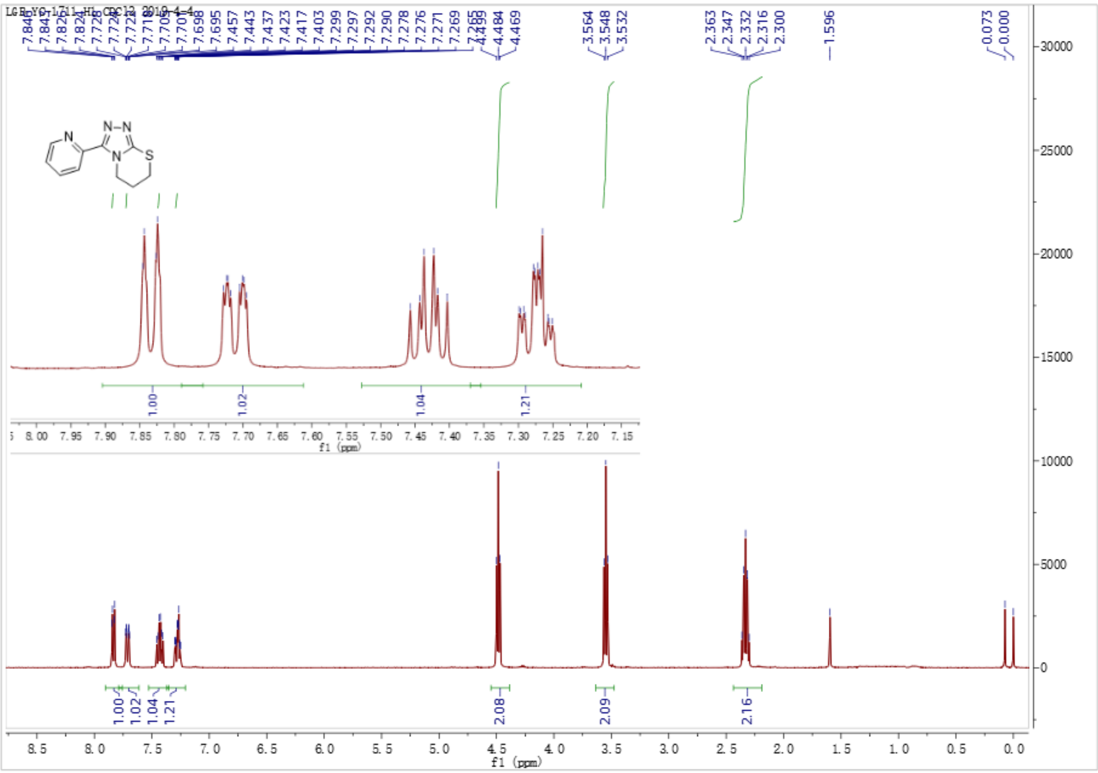

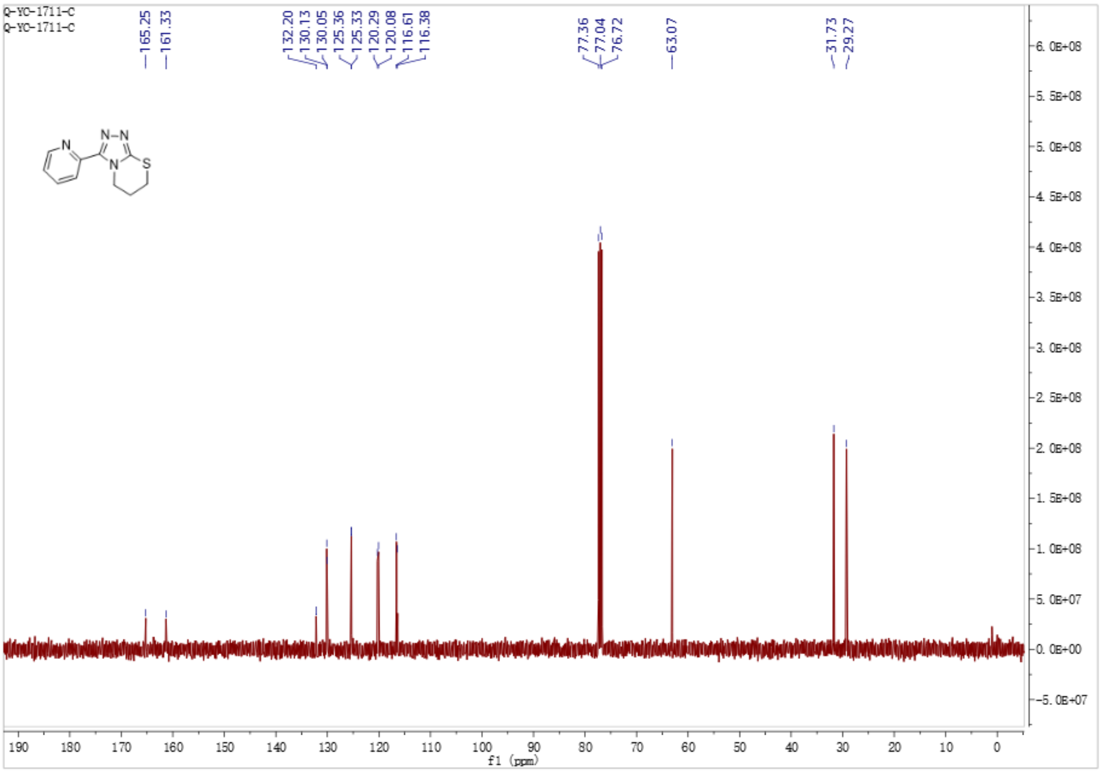

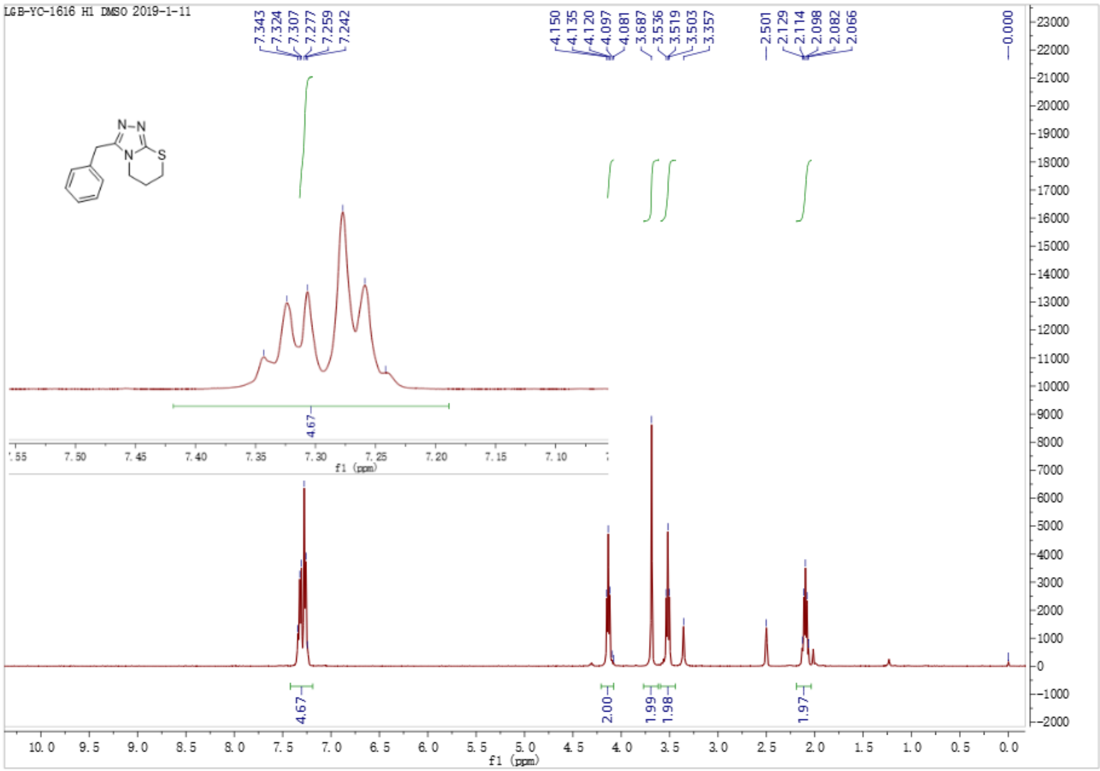
LGB-YC-1616 H1 DMSO 2019-1-11
7.343
7.324
7.307
7.277
7.259
7.242
4.150
4.135
4.120
4.097
4.081
3.687
3.536
3.519
3.503
3.357
2.501
2.129
2.114
2.098
2.082
2.066
0.000
4.67
2.00
1.99
1.98
1.97
f1 (ppm)

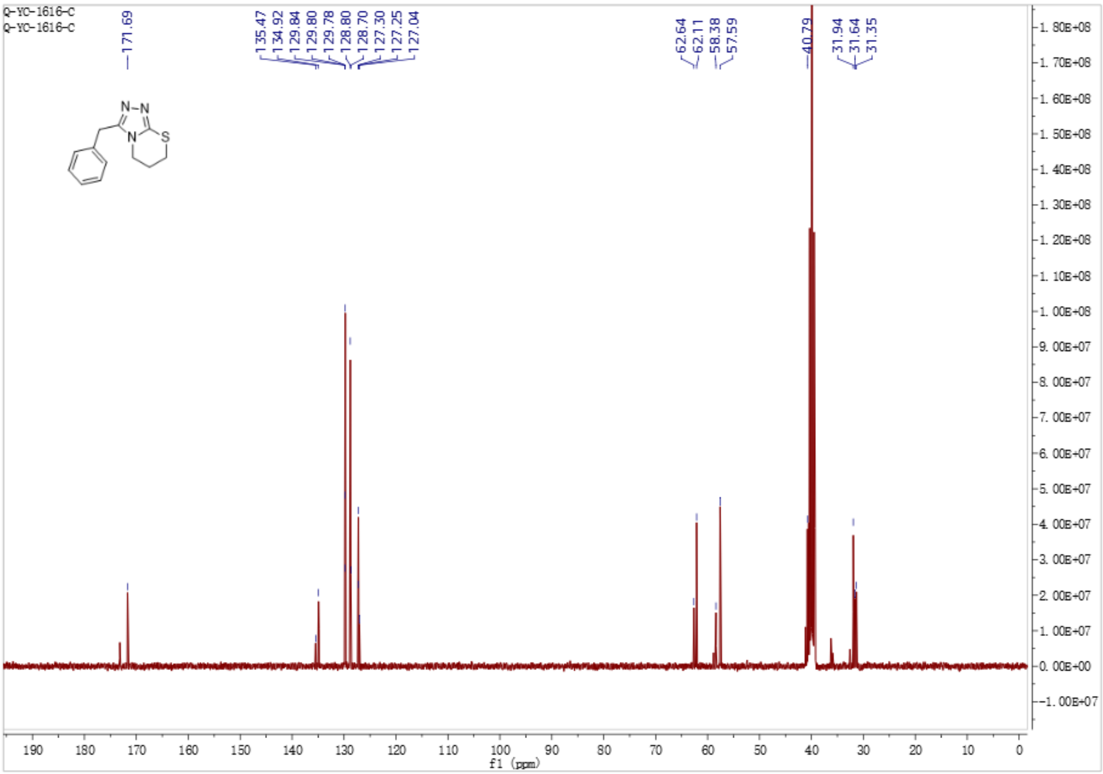
Q-YC-1616-C
171.69
135.47
134.92
129.84
129.80
129.78
128.80
128.70
127.30
127.25
127.04
62.64
62.11
58.38
57.59
40.79
31.94
31.64
31.35
f1 (ppm)

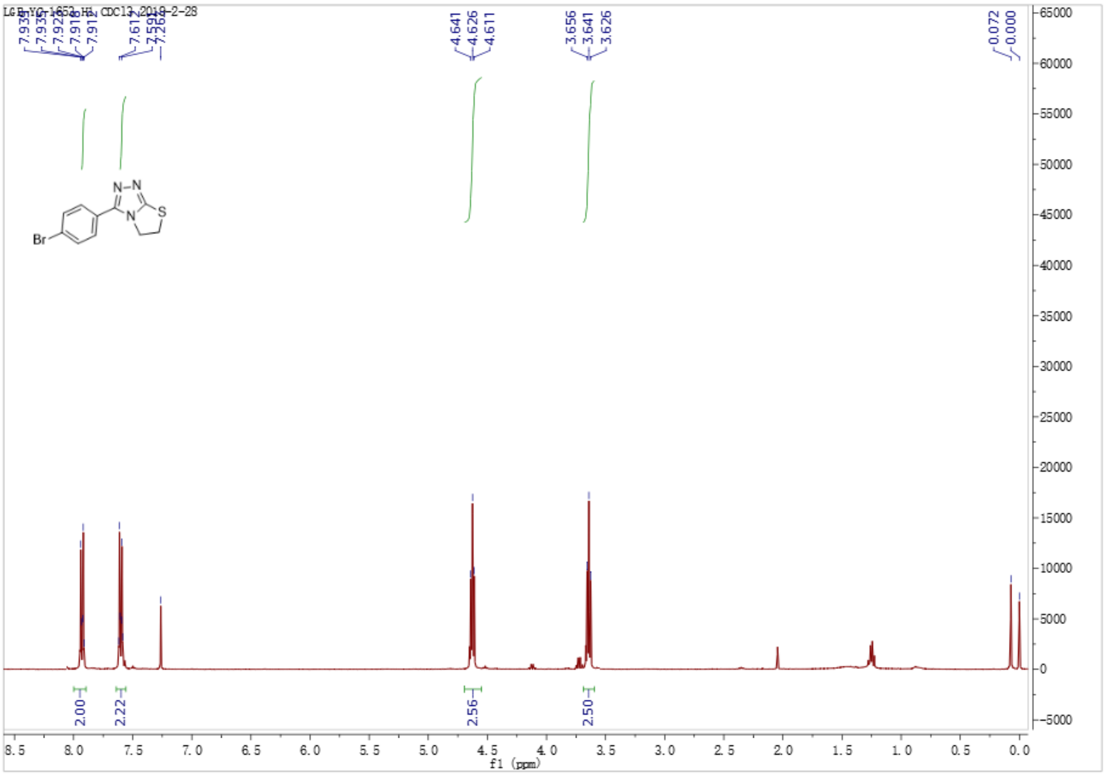
7.939
7.935
7.928
7.918
7.912
7.612
7.591
7.264
4.641
4.626
4.611
3.656
3.641
3.626
0.072
0.000
2.00
2.22
2.56
2.50
f1 (ppm)
N–N
S
N
Br

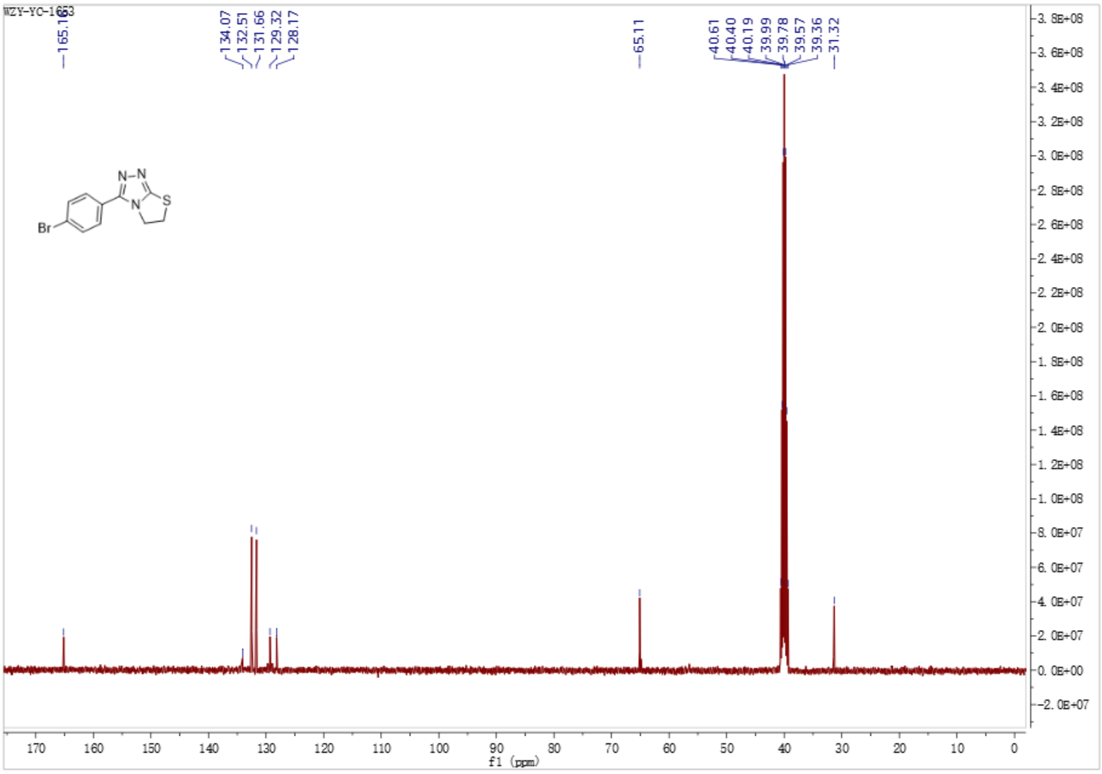
165.18
134.07
132.51
131.66
129.32
128.17
65.11
40.61
40.40
40.19
39.99
39.78
39.57
39.36
31.32
f1 (ppm)
N–N
S
N
Br

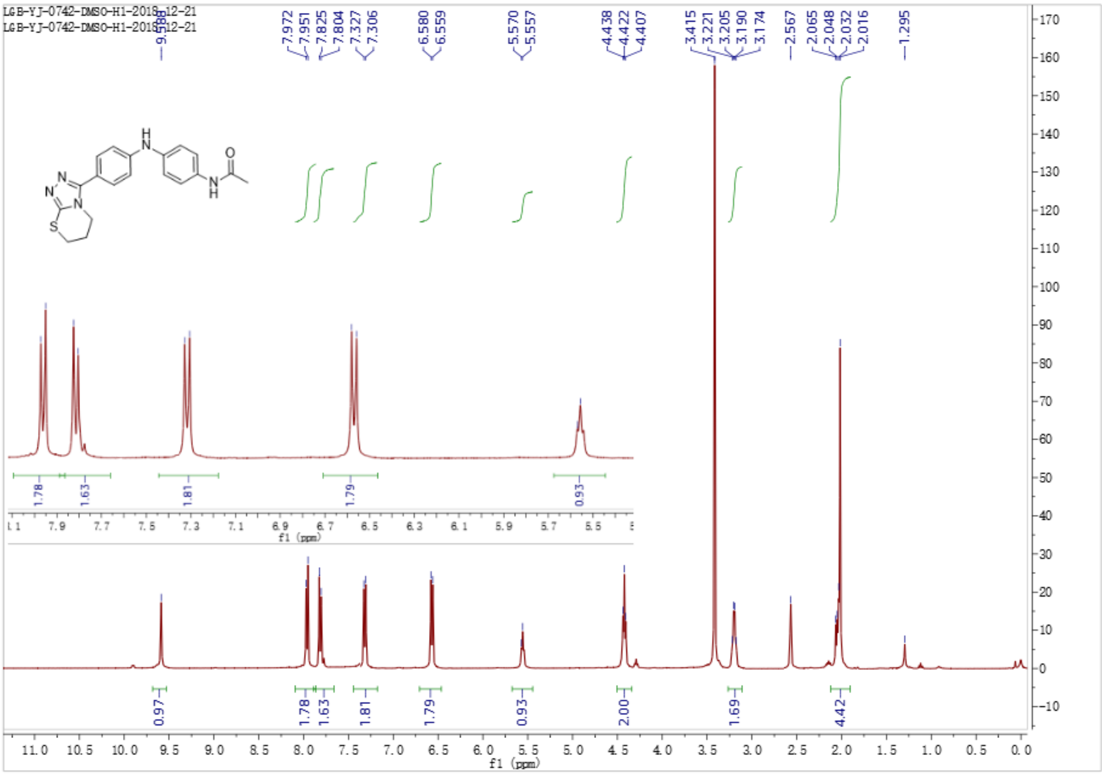
LGB-YJ-0742-DMSO-H1-2018-12-21
f1 (ppm)

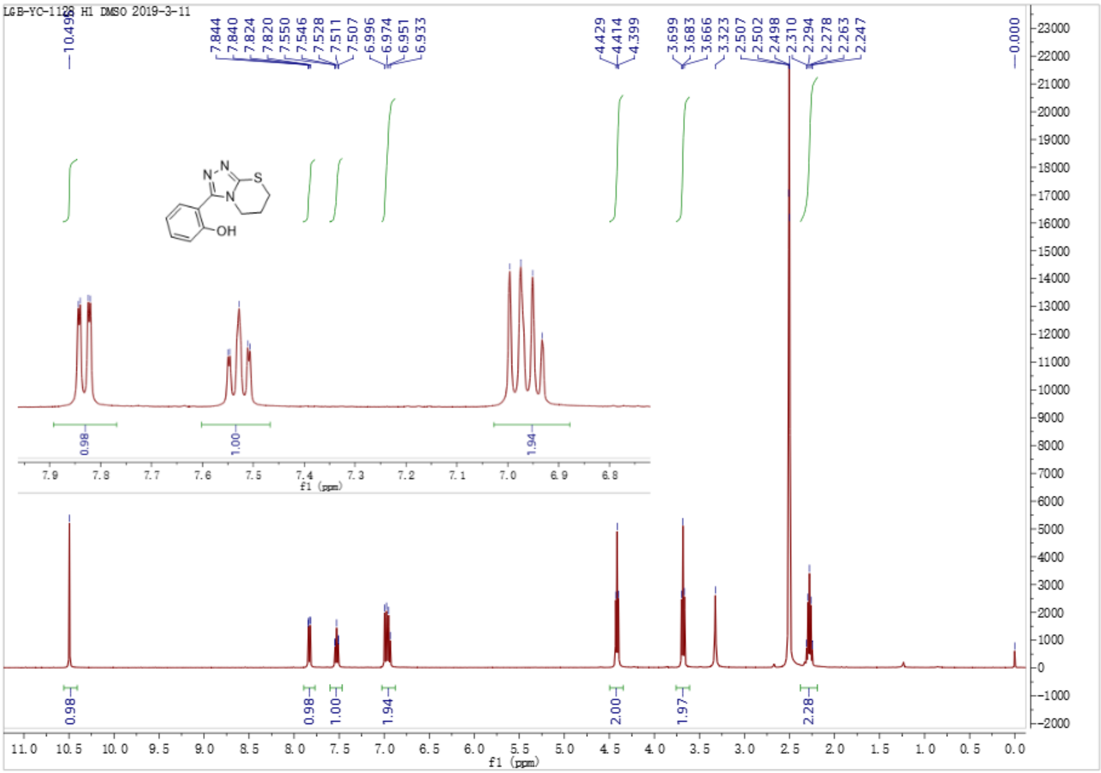
LGB-YC-1128 H1 DMSO 2019-3-11
OH
f1 (ppm)

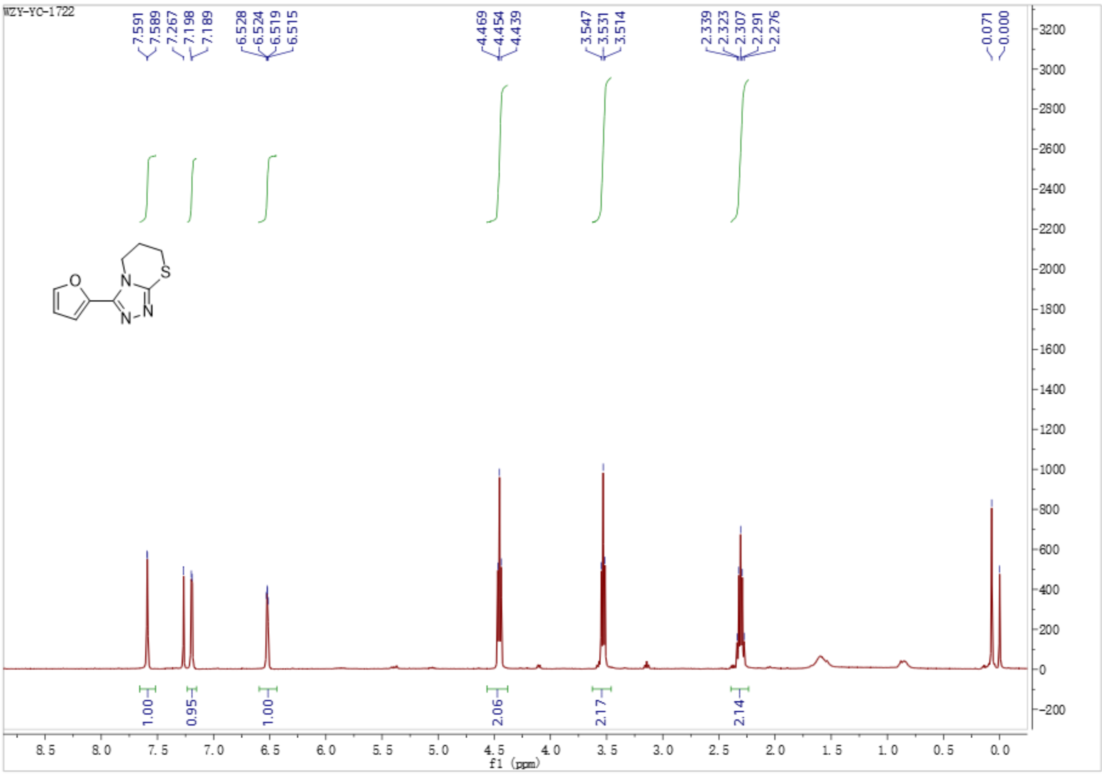

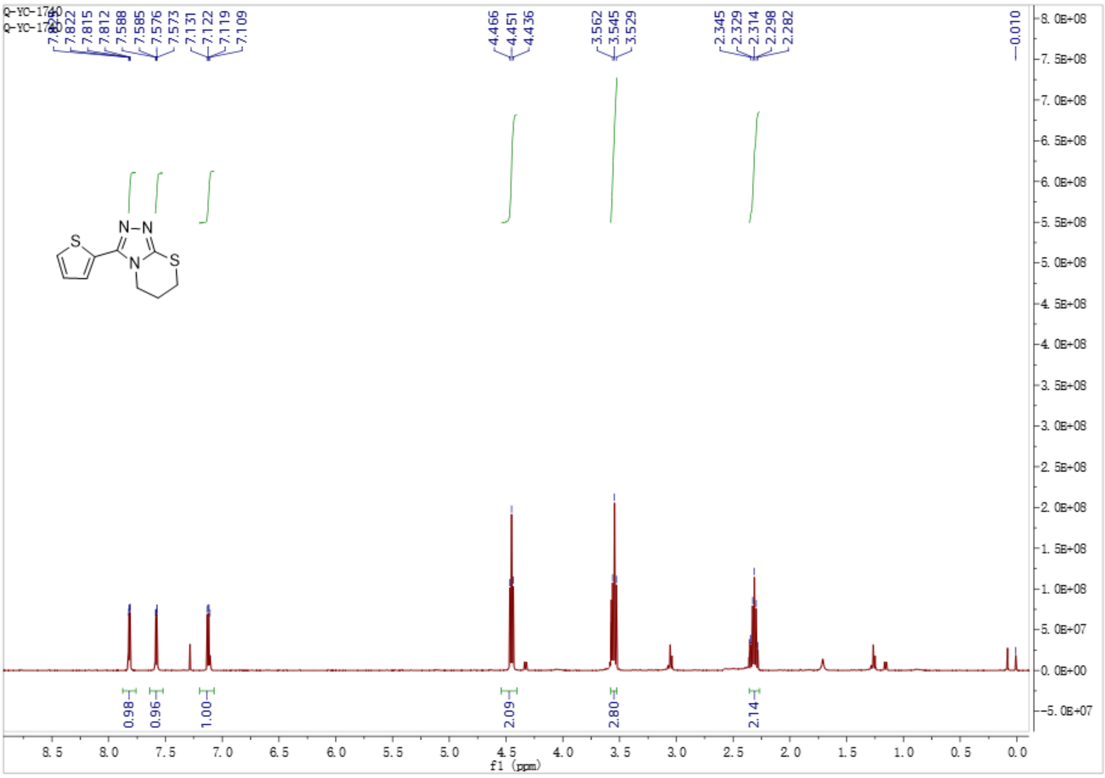

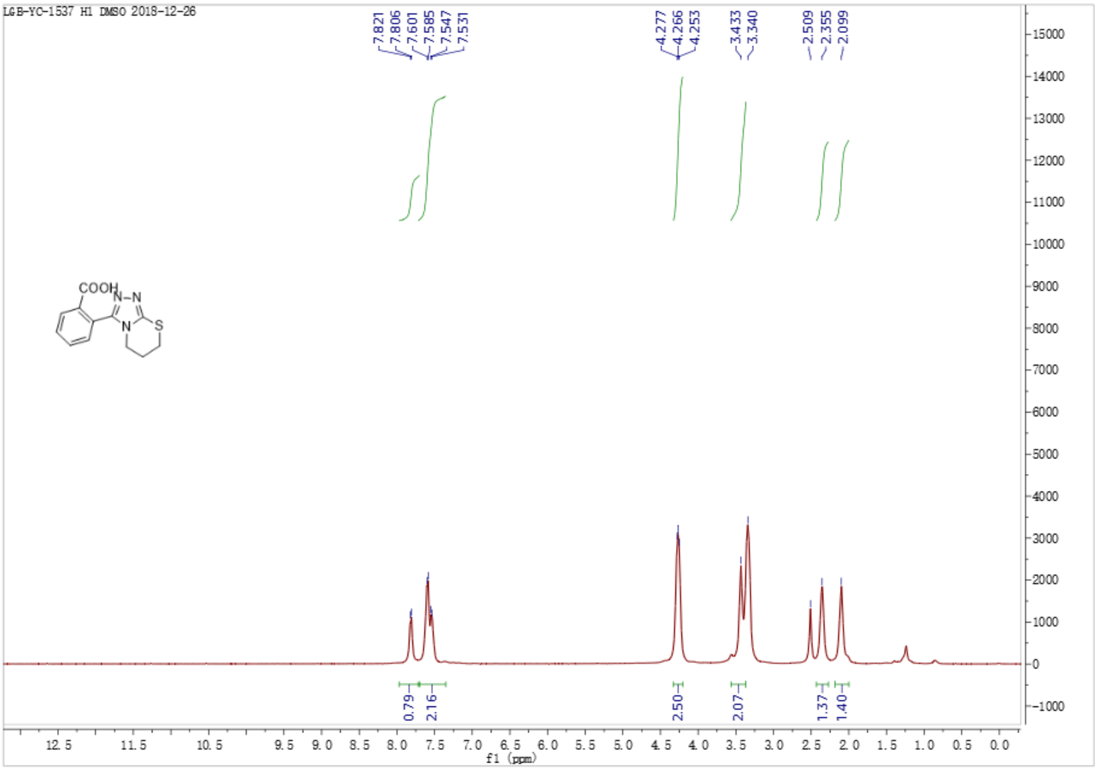
LGB-YC-1537 H1 DMSO 2018-12-26
7.821
7.806
7.601
7.585
7.547
7.531
4.277
4.266
4.253
3.433
3.340
2.509
2.355
2.099
COOH
N-N
N
S
0.79
2.16
2.50
2.07
1.37
1.40
f1 (ppm)

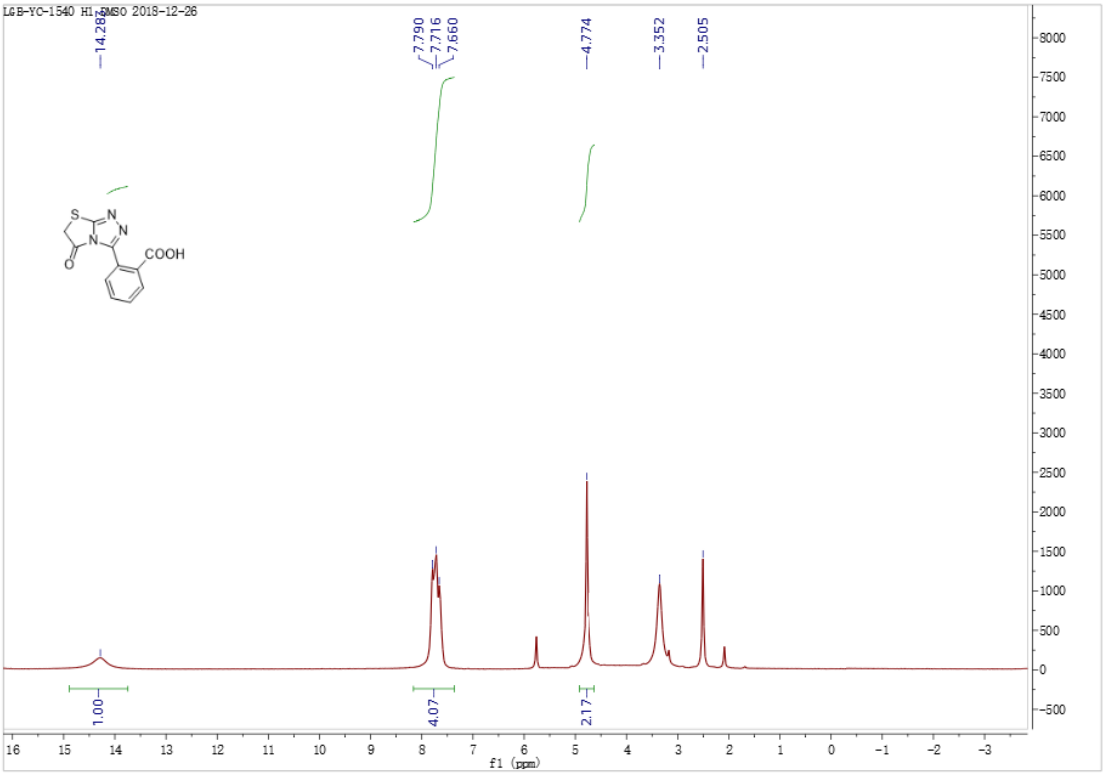
LGB-YC-1540 H1 DMSO 2018-12-26
14.28
7.790
7.716
7.660
4.774
3.352
2.505
S
N
N
N
O
COOH
1.00
4.07
2.17
f1 (ppm)
